# An unbiased AAV-STARR-seq screen revealing the enhancer activity map of genomic regions in the mouse brain in vivo

**DOI:** 10.1038/s41598-023-33448-w

**Published:** 2023-04-25

**Authors:** Ya-Chien Chan, Eike Kienle, Martin Oti, Antonella Di Liddo, Maria Mendez-Lago, Dominik F. Aschauer, Manuel Peter, Michaela Pagani, Cosmas Arnold, Andreas Vonderheit, Christian Schön, Sebastian Kreuz, Alexander Stark, Simon Rumpel

**Affiliations:** 1 Institute of Physiology, Focus Program Translational Neurosciences, University Medical Center, Johannes Gutenberg University Mainz, Mainz, Germany; 2 Institute of Molecular Biology GmbH (IMB), Mainz, Germany; 3 Research Institute of Molecular Pathology (IMP), Vienna Biocenter (VBC), Vienna, Austria; 4 Research Beyond Borders, Boehringer Ingelheim Pharma GmbH & Co. KG, Biberach an Der Riß, Germany; 5 Medical University of Vienna, Vienna BioCenter (VBC), 1030 Vienna, Austria; 6 Present Address: Global Computational Biology and Digital Sciences, Boehringer Ingelheim Pharma GmbH & Co. KG, Biberach an Der Riß, Germany; 7 Present Address: Department of Stem Cell and Regenerative Biology, Harvard University, Cambridge, MA USA; 8 Present Address: CeMM Research Center for Molecular Medicine, Austrian Academy of Sciences, Vienna, Austria

**Keywords:** Molecular biology, Neuroscience

## Abstract

Enhancers are important cis-regulatory elements controlling cell-type specific expression patterns of genes. Furthermore, combinations of enhancers and minimal promoters are utilized to construct small, artificial promoters for gene delivery vectors. Large-scale functional screening methodology to construct genomic maps of enhancer activities has been successfully established in cultured cell lines, however, not yet applied to terminally differentiated cells and tissues in a living animal. Here, we transposed the Self-Transcribing Active Regulatory Region Sequencing (STARR-seq) technique to the mouse brain using adeno-associated-viruses (AAV) for the delivery of a highly complex screening library tiling entire genomic regions and covering in total 3 Mb of the mouse genome. We identified 483 sequences with enhancer activity, including sequences that were not predicted by DNA accessibility or histone marks. Characterizing the expression patterns of fluorescent reporters controlled by nine candidate sequences, we observed differential expression patterns also in sparse cell types. Together, our study provides an entry point for the unbiased study of enhancer activities in organisms during health and disease.

The identity and function of a cell is to a large part determined by the set of expressed genes. In recent years the systematic analysis of single-cell expression patterns has revolutionized the taxonomy of cell types in various organs, including the brain, and has led to the delineation of over hundred distinct types in the cortex alone^1,2^. The emergence of a cell-type specific gene expression pattern is mediated by diverse processes among which the interplay of gene promoters and enhancers has been identified as a major mechanism^3–6^.

For basic research, as much as for future therapeutic approaches, it is of interest to gain genetic access to specific cell types. Here, viral vectors and in particular adeno-associated-viruses (AAV) have become a widely-used tool for gene delivery. Harnessing the selective transcriptional activity provided by specific promoter elements can target the expression of genes of interest to desired cell types^7^. In recent years, the combination of a core promoter together with enhancers active in different cell types has been successfully employed to drive transgene expression in select subpopulations of cortical neurons and to expand the still limited repertoire of available promoter elements for AAV^8–14^.

The identification of enhancers often relies on indirect measures, such as DNA conservation or epigenetic signatures such as open chromatin accessibility or histone tail modifications^15–18^. Traditionally, laborious and time-consuming reporter assays have been used to directly test if a DNA sequence acts as a functional enhancer^19,20^. To enable screens on a larger scale, the Massively Parallel Reporter Assay (MPRA) approach was developed allowing the simultaneous screening of thousands of candidate enhancers by pairing them with DNA barcodes, which are incorporated into the associated transcripts and used as activity readouts.^21,22^. In addition to cell lines, this technique has been recently extended for use in vivo by packaging MPRA constructs into AAV capsids^23,24^. MPRA has been extended to the single nucleus level in vivo, by combining it with single nucleus RNA-sequencing in the Paralleled Enhancer Single Cell Assay (PESCA) approach^25^. In contrast to epigenetic assays, MPRA screens directly measure enhancer activity, i.e. their ability to activate gene transcription^26^.

A drawback of MPRA is that the coupling of candidate enhancers with DNA barcodes complicates the generation of screening libraries, and does not readily scale to millions of candidate sequences. To overcome this limitation, the STARR-seq technique was developed^27^. This is a variant of the MPRA approach that incorporates genomic fragments downstream of a minimal promoter. The fragments containing enhancers can interact with the minimal promoter and drive the expression of an mRNA transcript that includes its own sequence. This eliminates the need for barcodes, simplifying library generation and enables the scaling to genome-wide screens of entire mammalian genomes^28–30^. Consequently, STARR-seq provides an unbiased assay of enhancer activity in large-scale libraries created from DNA fragments without the necessity to pre-select candidates. So far, unbiased large-scale STARR-seq screens have only been carried out in cell culture systems.

As with the original MPRA technique, a STARR-seq enhancer screen using AAV as a vector has been performed in vivo in mice^31^, however, both this and previous in vivo MPRA-based screens^24,25^ assayed a pre-selected panel of a few hundred to up to 3,500 candidate sequences. The power of STARR-seq approaches in cultured cells, however, was to leverage its efficacy to tile the entire genome, or at least larger genomic regions, in an unbiased manner to create maps of enhancer activities.

Here, we tested whether STARR-seq can be effectively used to screen high complexity genomic libraries covering entire genomic loci surrounding genes of interest in mammals in vivo. We describe an unbiased STARR-seq screen in the brain of a living mouse using AAV capsids to deliver a screening library covering nine different regions of the mouse genome. Within these genomic regions, we identified 483 candidate enhancers in an unbiased fashion, several of which we validated with in vivo fluorescence reporter assays. We further analyzed two of the candidates in high detail, characterizing their target cell types. In summary, we demonstrate that the STARR-seq technique can be transposed for large-scale, unbiased enhancer screening in vivo using AAV (AAV-STARR-seq). We identify and validate several mouse brain enhancers, demonstrating its viability as an in vivo screening system for the development of small, artificial promoter elements.

## Results

### An AAV-STARR-seq screen in the mouse brain

**Figure 1:**
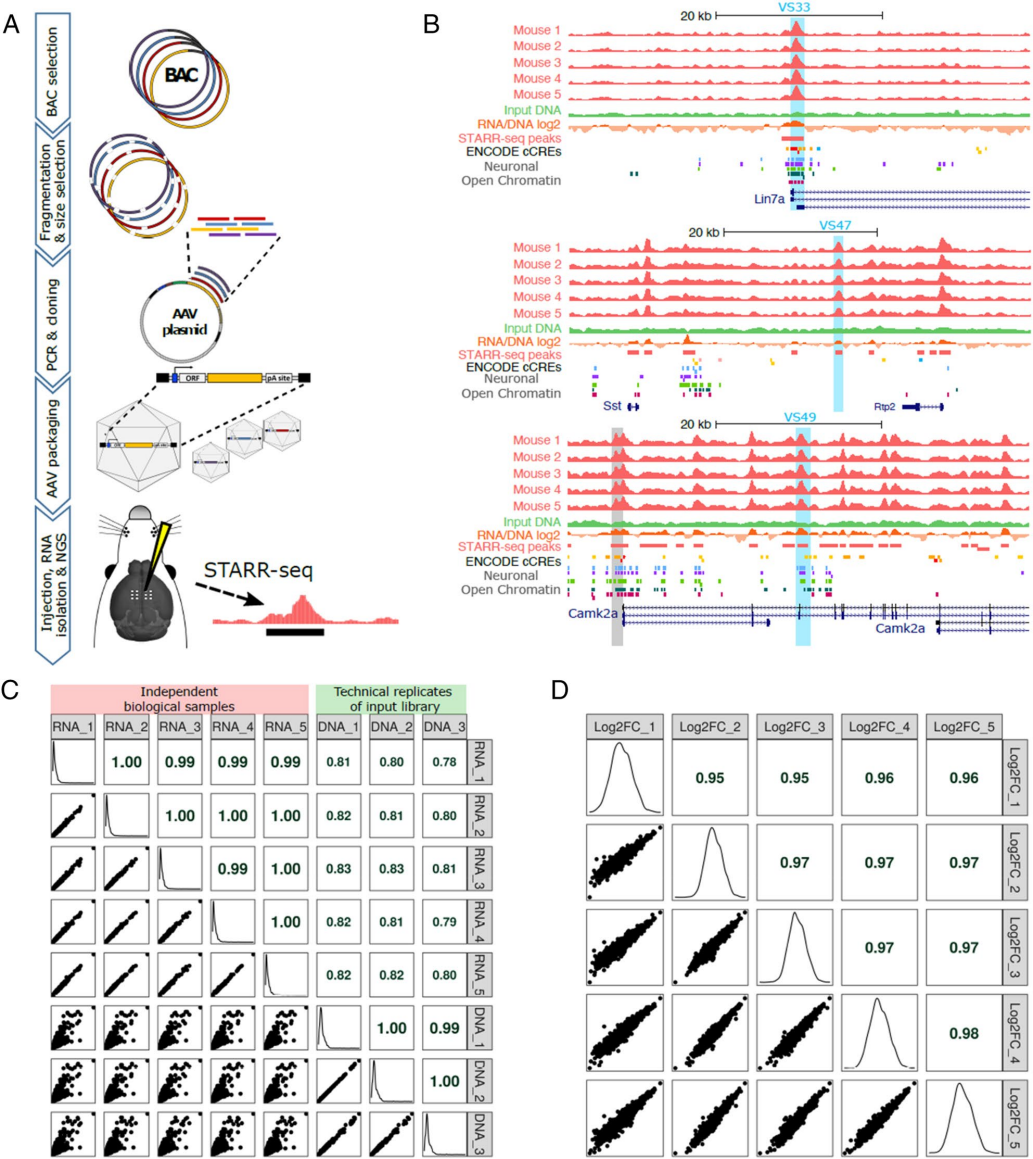
An AAV-STARR-seq screen in mouse brain. (**A**) Schematic illustrating AAV-STARR-seq screening procedure. (**B**) UCSC genome browser screenshots of three example regions, showing the STARR-seq RNA from 5 mice and the input library DNA (merged from the 3 technical replicates). The blue highlighted regions were further evaluated in this study. The grey highlighted region shows the 1.2 kb Camk2a promoter region from Dittgen et al.^61^ used in commercially available AAV2 constructs (Addgene plasmid # 22908). The ENCODE cCRE track shows enhancers (orange), promoters (red) and insulators (blue) identified by the ENCODE project in various mouse tissues and cell types. Neuronal Open Chromatin tracks are from Gray et al.^35^, for mouse visual cortex layer-specific excitatory neurons from layers 2/3 (light blue), 4 (purple), 5 (light green), and 6 (dark green), and GABAergic neurons from all layers (dark red). (**C**) Pairwise scatter plots and Pearson correlation coefficients for all independent biological RNA samples from the five mice and the three technical replicates from the AAV-STARR-seq library DNA used as input for the screen, based on 1 kb bins tiled across the BAC regions. The diagonal plots show the expression distributions of the 1 kb bins per sample. (**D**) Pairwise scatter plots and Pearson correlation coefficients for the STARR-seq signal (RNA/input DNA log2 fold change) across all mice.

In order to perform an AAV-STARR-seq screen of entire genomic regions in vivo, we nominated a set of genes known for strong and constitutive expression in the mouse brain e.g. Ca_2 _+ /calmodulin-dependent protein kinase II (*CamkII*), Glial fibrillary acidic protein (*Gfap*) or somatostatin (*Sst*) (Supplemental Table 1). We obtained bacterial artificial chromosomes (BACs) covering the selected genes that in total spanned about 3 Mb of the mouse genome (~ 0.1%) (Supplemental Table 1). Following sonication and size selection (~ 700 bp), we cloned the genomic fragments into an AAV-STARR-seq screening plasmid downstream of the 4.26 minimal promoter using InFusion cloning (Fig. 1A)^27^. In this configuration, genomic fragments displaying enhancer activity can interact with the minimal promoter and drive expression of transcripts including their own sequence. This makes them detectable at the mRNA level using next generation sequencing methodology^27^. After batch cloning of fragments, the AAV-STARR-seq library plasmid containing AAV2 inverted terminal repeat (ITR) was packaged into AAV8 capsids. AAV8 capsids have been previously shown to show enable broad and efficient transduction of major neuronal and glial cell classes in the mouse brain^32^. Formation of double-stranded DNA and fast transcription of mRNA in the mouse brain was facilitated as one of the ITRs was lacking a terminal resolution site^32,33^. Coverage of the BAC regions, as estimated by sequencing the packaged screening library, was high with the majority of nucleotides in the BAC regions being covered at a depth of at least 100-fold (Supplemental Fig. S1A). The virus suspension was stereotactically injected in 12 nearby sites covering a major part of the dorsal neocortex in 5 mice (see Methods). After an incubation period of 7 days, the mice were sacrificed and the injected region was removed and total RNA was extracted for STARR-seq RNA processing. Self-complementing AAV vectors, as used for the screening library, enable expression in the mouse brain already one week after injection^32^.

Following sequencing and mapping of reads to the reference genome, we found that the RNA reads obtained from the screen showed a characteristic enrichment for select genomic loci indicative of enhancer activity reminiscent of previous STARR-seq results obtained in cultured cells (Fig. 1B). In individual mice we identified about 500,000 unique transcripts (Supplemental Table 2). Reproducibility of AAV library-derived mRNA across all independently processed and analyzed mice was remarkably high, indicating an efficient delivery of the screening library to the brain tissue in individual mice (Pearson correlation coefficient (PCC) >  = 0.99) (Fig. 1C). We also observed a high degree of correlation with the input DNA library (PCC = 0.82 to 0.86), suggesting a basal level of background transcription of the AAV library sequences, regardless of enhancer activity. Coverage levels of the RNA across the BAC regions were substantially lower than the input DNA library, but still most nucleotides were covered at least tenfold (Supplemental Fig. S1A). Importantly, the observed levels of transcriptional activity of the library sequences (RNA/input DNA) were also highly reproducible across mice (PCC = 0.95–0.98) (Fig. 1D). Given that STARR-seq assays differences in transcriptional activity between genomic regions, removal of duplicated sequences from the data lead to higher correlation between RNA and input DNA samples (PCC = 0.92–0.94) (Supplemental Fig. S1B), while the reproducibility of transcriptional activity across mice dropped slightly (PCC = 0.93–0.96) (Supplemental Fig. S1C).

### Identification of candidate enhancer regions

**Figure 2:**
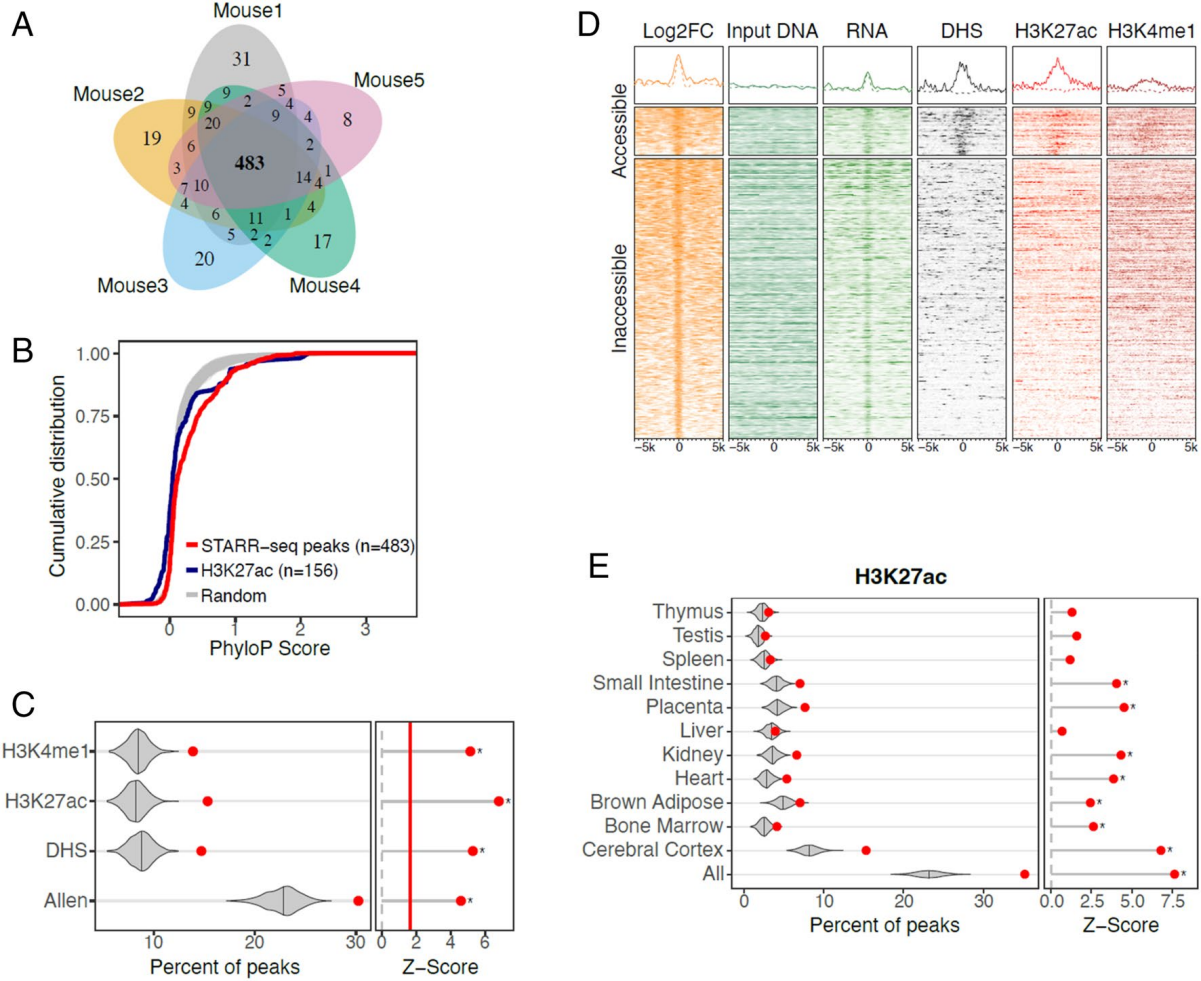
Candidate enhancer identification. (**A**) Reproducibility across mice of the RNA/input DNA peaks called by the STARRPeaker algorithm. Plotted is the number of mice in which a given peak has been identified. Note, that the majority of peaks were identified in all five mice independently. (**B**) Cumulative distribution of PhyloP evolutionary conservation scores of the candidate enhancer peaks (red line) compared with 1000 randomly shuffled control peak sets (grey lines), indicating an increased conservation of AAV-STARR-seq peaks. For comparison, the cumulative distribution of H3K27ac marks within the screened genomic regions is shown (blue line). (**C**) Overlap of candidate enhancer peaks with active enhancer-associated histone marks (H3K4me1 and H3K27ac) and accessible chromatin regions in adult mouse brain cortex (red dots), compared with 1000 shuffled controls (violin plots). The right panel lollipop plot shows the Z-scores of the real overlap based on fitting a normal distribution to the random controls. Asterisks indicate significant differences based on empirical *p* < 0.05. DHS: Dnase1 Hypersensitive Site dataset from the ENCODE project; Allen: Pooled neuron subtype-specific ATAC-seq peak datasets from the Allen Brain Atlas. (**D**) Heatmap showing the input DNA, merged RNA and the corresponding RNA/DNA log2 fold change signal around the STARR-seq peaks (rows), as well as the accessible chromatin, H3K27ac and H3K4me1 histone mark signal from adult mouse cortex. The heatmap is clustered into STARR-seq peaks overlapping accessible chromatin peaks (above) and non-overlapping peaks (below), and sorted by RNA/DNA log2 fold change (STARR column). (**E**) Overlap of candidate enhancer peaks with H3K27ac active enhancer-associated histone marks in several adult mouse organs. Violin and lollipop plots are created as in (C). Asterisks indicate significant differences based on empirical *p* < 0.05.

Using the AAV-packaged input DNA as control, we identified 483 candidate enhancers as peaks that were independently detected in all five mice, using the STARRPeaker program^34^ (Fig. 2A). The fact that about 66% of all the peaks that have been detected in at least one mouse, have been detected in five mice independently indicates a high level of reproducibility. Similar results were obtained using the MACS2 peak caller program, albeit with slightly lower reproducibility across mice. We considered all peaks detected by STARRPeaker regardless of peak score threshold, as the peak count saturated with decreasing threshold (Supplemental Fig. S2B). The genomic regions identified in the AAV-STARR-seq screen had higher PhyloP evolutionary conservation scores than shuffled control regions (Wilcoxon rank sum test: *p* < 0.001) (Fig. 2B). Furthermore, they showed an overall enrichment for mouse brain open chromatin regions and active enhancer-associated histone marks from the ENCODE project^15^ and Allen Brain Institute^35^ (Fig. 2C, Supplemental Fig. S2C,D). However, consistent with previous findings^28–30^, we observed that only a subset of peaks overlapped with open chromatin regions in mouse cerebral cortex and were also associated with the H3K27ac and H3K4me1 active enhancer histone marks (Fig. 2D). Many identified peaks did not overlap with open chromatin regions and active enhancer marks (Fig. 2D). This suggests that these genomic sequences have the potential to act as enhancers, but are inactivated in the mouse brain by epigenetic mechanisms.

To gain further insight, we considered the AAV-STARR-seq peaks from the cerebral cortex and analyzed if they showed enrichment for the H3K27ac active enhancer histone mark^36^ that has been detected in other mouse organs, but not in the brain (Fig. 2E). We found an increased overlap with enhancer marks for non-brain organs, albeit at a lower level than for the cerebral cortex. This further corroborates that, enhancers endogenously active in other mouse organs, but not in mouse brain, can become active in cerebral cortex when delivered in an episomal context using AAV.

We did not identify any clearly enriched DNA motifs, apart from a few low complexity sequences, which may be related to the small number of peaks detected. In contrast to the enhancer-related epigenetic marks, repetitive genomic elements were not enriched amongst the STARR-seq peaks, and retrotransposon classes like LINEs and endogenous retroviruses were even depleted (Supplemental Fig. S2E). Consistent with this, these retrotransposon classes were enriched in regions that had relatively low RNA levels compared to input DNA levels (Supplemental Fig. S2F).

Together, our bioinformatic analyses indicated that genomic sites displaying increased transcriptional activity in the AAV-STARR-seq screen show an overall enrichment for established enhancer marks in the brain, but also other organs.

### Multiplexed validation of candidate sequences using fluorescence reporters

**Figure 3:**
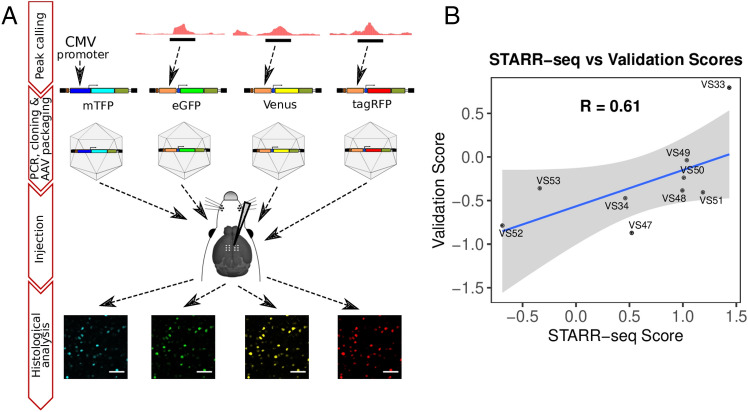
AAV-STARR-seq validation procedure. (**A**) Schematic illustrating AAV-STARR-seq validation procedure. (**B**) Scatterplot of AAV-STARR-seq screen signal (RNA/input DNA log2 fold change, averaged across mice) and fluorescence microscopy-based validation signal (log10 median relative fluorescence strength compared to CMV promoter; see Fig. 4) for 9 validation candidates with varying predicted enhancer activity.

Brain tissue is complex in its composition is formed from over hundred distinct cell types^1,2^. The AAV-STARR-seq screen was based on the mRNA extracted from a homogenized cortical tissue sample. As such, it does not provide further information if the activity in the screen arises from a broad expression pattern or is restricted to few cell types. To shed light on this matter and to independently validate the functionally identified candidate enhancers, we chose nine genomic sequences (validation sequences, VS) that were covered in the screen and that were selected to cover the whole range of activities from very low levels to very high levels, indicating candidate enhancers. Thus, the nine validation sequences included peaks of enhancer activity consistent with epigenetic marks, peaks of enhancer activity that were not predicted by epigenetic marks and also regions that did not show enhancer activity in the screen (Supplemental Fig. S3). We conducted an in vivo validation experiment using a multiplexed fluorescence assay. Here, the validation sequences were cloned upstream of the 4.26 minimal promoter controlling the expression of a fluorescent reporter protein. This follows the typical design of an expression construct, where the gene of interest is positioned downstream of the control element driving transcription. It is often used for validation of enhancer elements as enhancers typically show similar activity levels in either the upstream or downstream configuration to a minimal promoter^37^. The size chosen for the validation sequences was between 1.2 and 1.5 kb as conventional rAAV constructs were used with a maximum genome size of 4.7 kb between the ITRs that can be packaged into AAV. For most validation sequences containing a peak in the AAV-STARR-seq screen (except VS52 and VS53), the genomic flanking regions were included and as such covered the full peaks which were typically larger than the ~ 700 bp fragments used in the screen. In order to keep the sequence length constant between the ITRs a stuffer sequence was cloned between the 5’ITR and the VS. Furthermore, it has been previously shown that ITRs located close to the reporter gene can have promoter function and drive expression alone^38,39^. The stuffer sequence was also inserted to counteract such a potential promoter function. We used four fluorescent reporter proteins (mTFP ‘blue’, EGFP ‘green’, Venus ‘yellow’ or tagRFP ‘red’) fused to a nuclear localization sequence to facilitate automated image processing (Fig. 3A). We designed a panel of four sets each containing four reporter constructs with the differently colored reporters. One set contained all four fluorescent reporters coupled to the CMV promoter, to serve as a normalization control to enable brightness comparisons across the different reporter fluorophores. The three other sets each consisted of three VSs and a CMV promoter control. The plasmids in each set were mixed in equimolar ratios and packaged together into AAV8 capsids. After adjustment of the viral titers the individual AAV preparations were injected into the auditory cortex, which shows a highly similar gene expression profile as the dorsal cortical regions used for the screens (see Methods). Given the longer expression times observed with conventional, non self-complementing rAAV and a readout based on the stable accumulation of fluorescent proteins using microscopy, we used an incubation time of three weeks, before mice were sacrificed and confocal images were taken from coronal sections of the auditory cortex. The activity of each VS was determined as the relative fluorescence of its reporter compared to that of the CMV-driven mTFP control reporter in the same preparation (see Methods). We observed a general positive correlation between the STARR-seq signal strengths (RNA/DNA log2 fold change of the mapped reads signal) and the relative fluorescence levels of the VS, but this correlation was relatively weak and did not reach statistical significance likely due to the limited number of tested sequences (Pearson’s correlation coefficient = 0.61, *p* = 0.08) (Fig. 3B).

**Figure 4:**
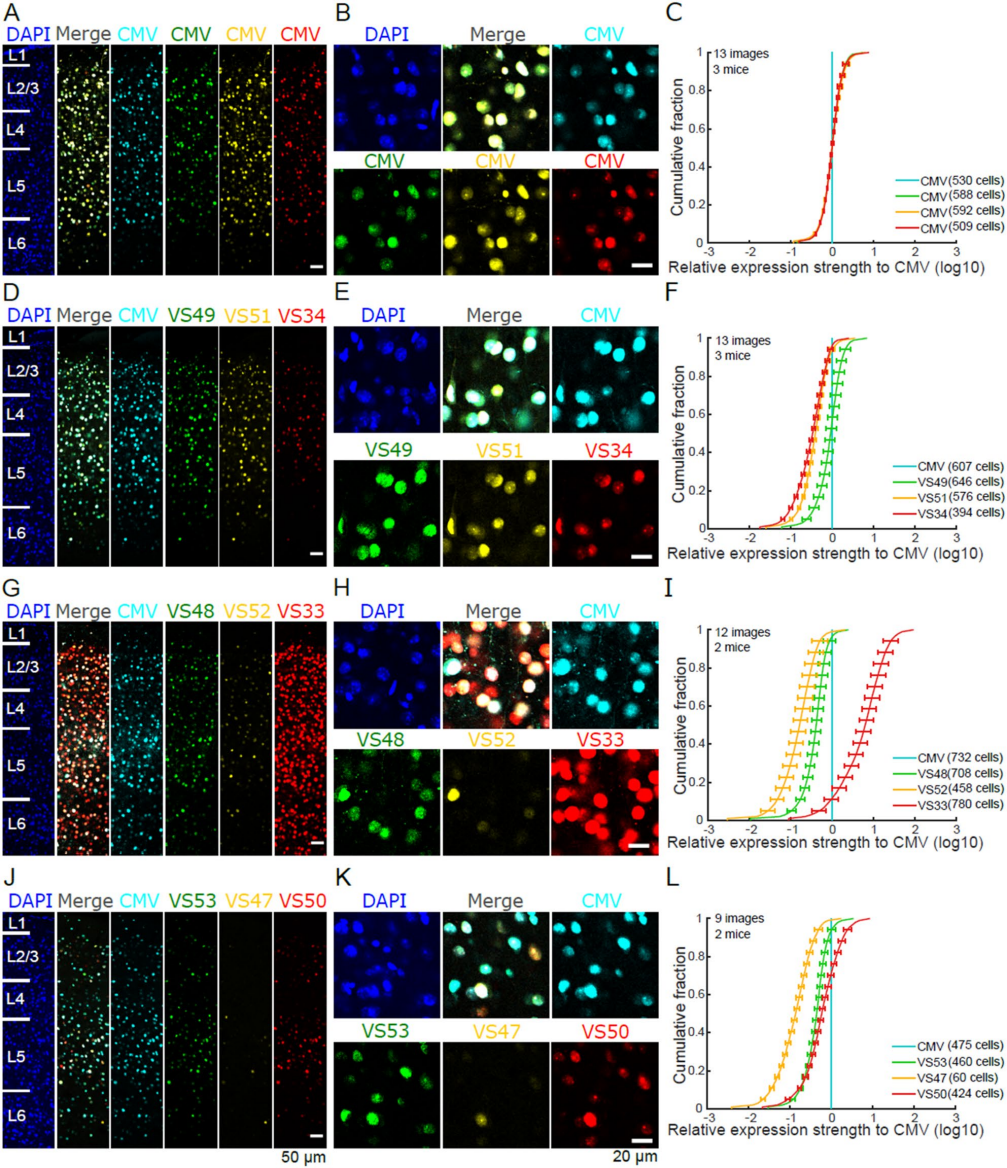
Multiplexed fluorescence microscopy of nine validation sequences in coronal sections of the auditory cortex. (**A**) Positive control with all fluorophore variants (mTFP, EGFP, Venus, and tagRFP) driven by CMV promoter. Six neocortical layers indicated on DAPI image. (**B**) Zoomed in view of example region. (**C**) Cumulative distribution across cells of the relative fluorescence intensity of EGFP, Venus, and tagRFP compared to mTFP (relative expression strength) for the CMV-driven control constructs (mean across images ± SD). (**D**–**L**) Analogous to (**A**–**C**), but for the nine validation sequences examined, divided into three sets of three sequences (**D**–**F**, **G**–**I**, **J**–**L**). Each set also contains a CMV promoter-driven mTFP reporter, to which all the others are compared (**F**, **I**, **L**). VS33 drives high expression levels (**G**, **H**, **I**).

When analyzing the expression patterns in the sections more closely, we observed that the nine tested validation sequences showed varying levels of fluorescence intensity and to some extent varying distribution across the cortical layers while the fluorescence signal in the all-CMV promoter-driven control set was highly similar for all fluorophores (Fig. 4). The observation from the control set indicates that individual cells were typically transduced by a similar number of the four constructs in each set. We therefore normalized the signal in the green, yellow and red fluorescence channel of individual cells to that in the blue channel, which was always controlled by the CMV promoter, in order to derive a quantitative measure of expression strength. Among the validation sequences, VS33 showed the strongest expression in both the STARR-seq screen and the fluorescence microscopy images at an almost ten-fold higher level than CMV. Most other validation sequences containing a clear peak in the AAV-STARR-seq showed an expression level comparable or somewhat weaker than the CMV promoter (VS34, VS48, VS49, VS50, VS51). Labeled nuclei were widely distributed across all cortical layers, but VS50 resulted in expression mostly in layer 5. Interestingly, VS47, despite being readily detected in the AAV-STARR-seq screen, showed an overall weak expression in the cortex that was restricted to very few cells in a given section. Two validation sequences that did not contain a clear AAV-STARR-seq peak (VS52 and VS53) showed expression weaker than CMV, that was nevertheless clearly detectable.

### Detailed characterization of the expression pattern of two candidates

We decided to characterize two hits from the AAV-STARR-seq screen more deeply and to test in how far the functionally identified enhancer candidates could be utilized to drive differential expression patterns. The first hit was the strong candidate enhancer VS33, which includes the transcription start site and downstream intronic regions of the *Lin7a* gene. The second hit was VS47, which only showed sparse expression in the multiplexed fluorescence assay. The corresponding AAV-STARR-seq peak is localized in the intergenic region between the *Sst* and *Rtp2* genes and, interestingly, did not overlap with enhancer-associated epigenetic marks or open chromatin regions. This sequence therefore unlikely would have been included in a targeted candidate enhancer screen.

**Figure 5:**
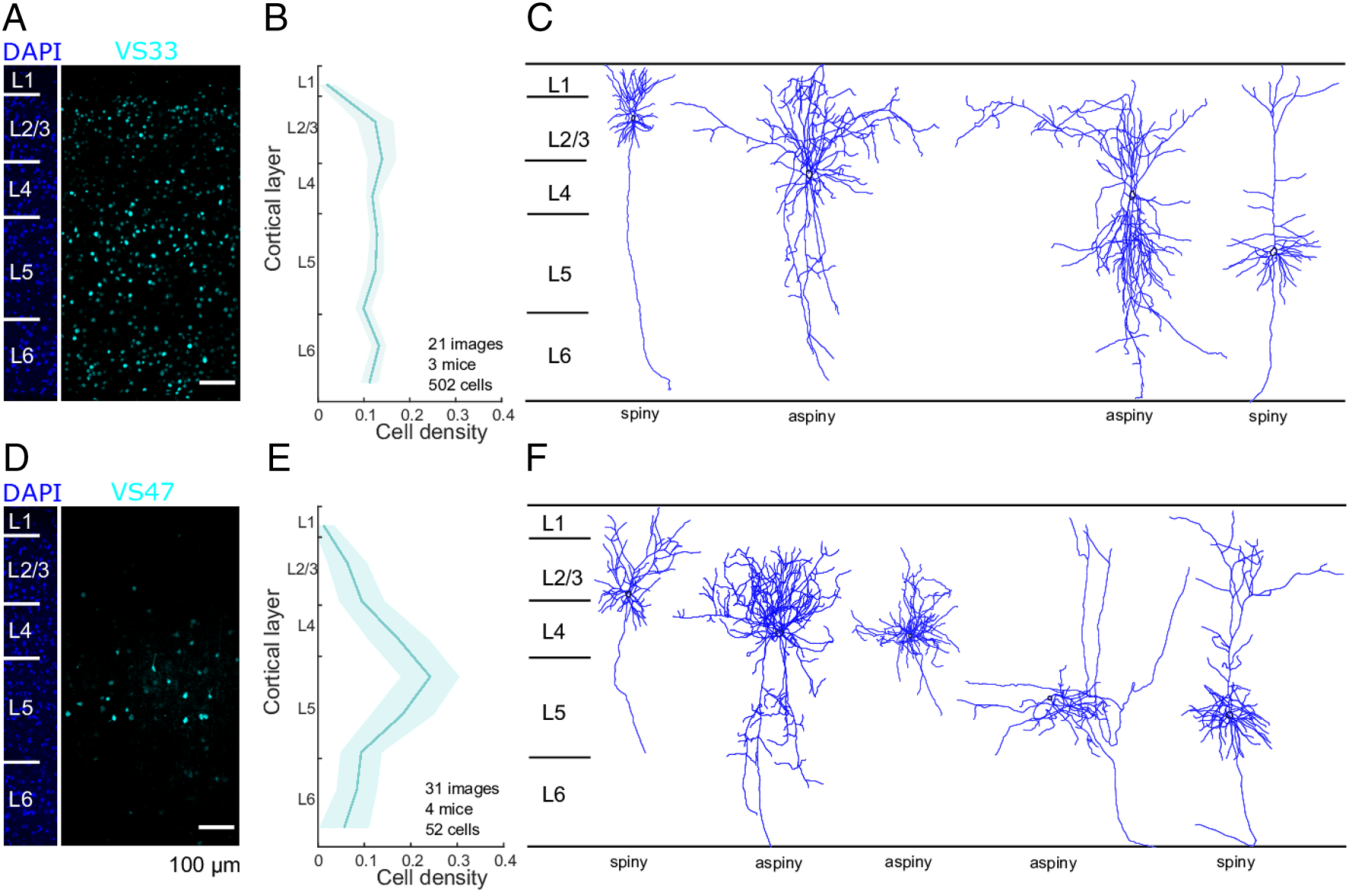
Cortical layer distribution and neuronal morphology of validation sequences VS33 and VS47 in coronal sections of the auditory cortex. (**A**, **D**) Example fluorescence microscopy image of a cortical slice depicting mTFP expression driven by VS33 (**A**) or VS47 (**D**). Six neocortical layers indicated on DAPI image. (**B**, **E**) Distribution of mTFP-expressing cells across cortical layers (mean across images ± SD) for VS33 (**B**) or VS47 (**E**). VS33 does not show layer-specificity, but VS47 drives expression mainly in layer5 of the cortex. (**C**, **F**) Example cell morphology tracings of neurons expressing EYFP after recombination byVS33-driven (**C**) or VS47-driven (**F**) Cre-recombinase. Both sequences drive expression in diverse morphological cell types.

We designed two constructs in which VS33 or VS47 were placed upstream of a 4.26 minimal promoter to drive a cre-recombinase and a nuclear-localized mTFP reporter and packaged them into AAV8 capsids. Coronal sections of the auditory cortex were analyzed using confocal microscopy after stereotactic injection and three weeks incubation time (Fig. 5). We first quantified the distribution of cells expressing the mTFP reporter across cortical layers. We observed a broad and strong expression of VS33 in most cortical layers except layer 1 (Fig. 5A,B). In contrast, VS47 was active in fewer cells, which were enriched in cortical layer 5 (Fig. 5D,E). To reveal the morphologies of the individual cells in which these candidate enhancers are active, we employed a sparse labeling approach and diluted the above-mentioned VS33 and VS47 Cre-mTFP constructs and mixed them with a cre-recombinase dependent AAV9-Ef1a-DIO-EYFP reporter virus leading to a cytoplasmic label of the entire cell. After stereotactic injection and three weeks of incubation, we took confocal image stacks in 500 µm thick, optically cleared coronal sections of the auditory cortex. We reconstructed the morphology of several cells for each candidate enhancer and found that the vast majority showed neuronal morphologies (Fig. 5C,F). Both VS33 and VS47 drove expression in spiny and aspiny neurons with a variety of different morphologies consistent with pyramidal- and interneuron-like identities.

**Figure 6:**
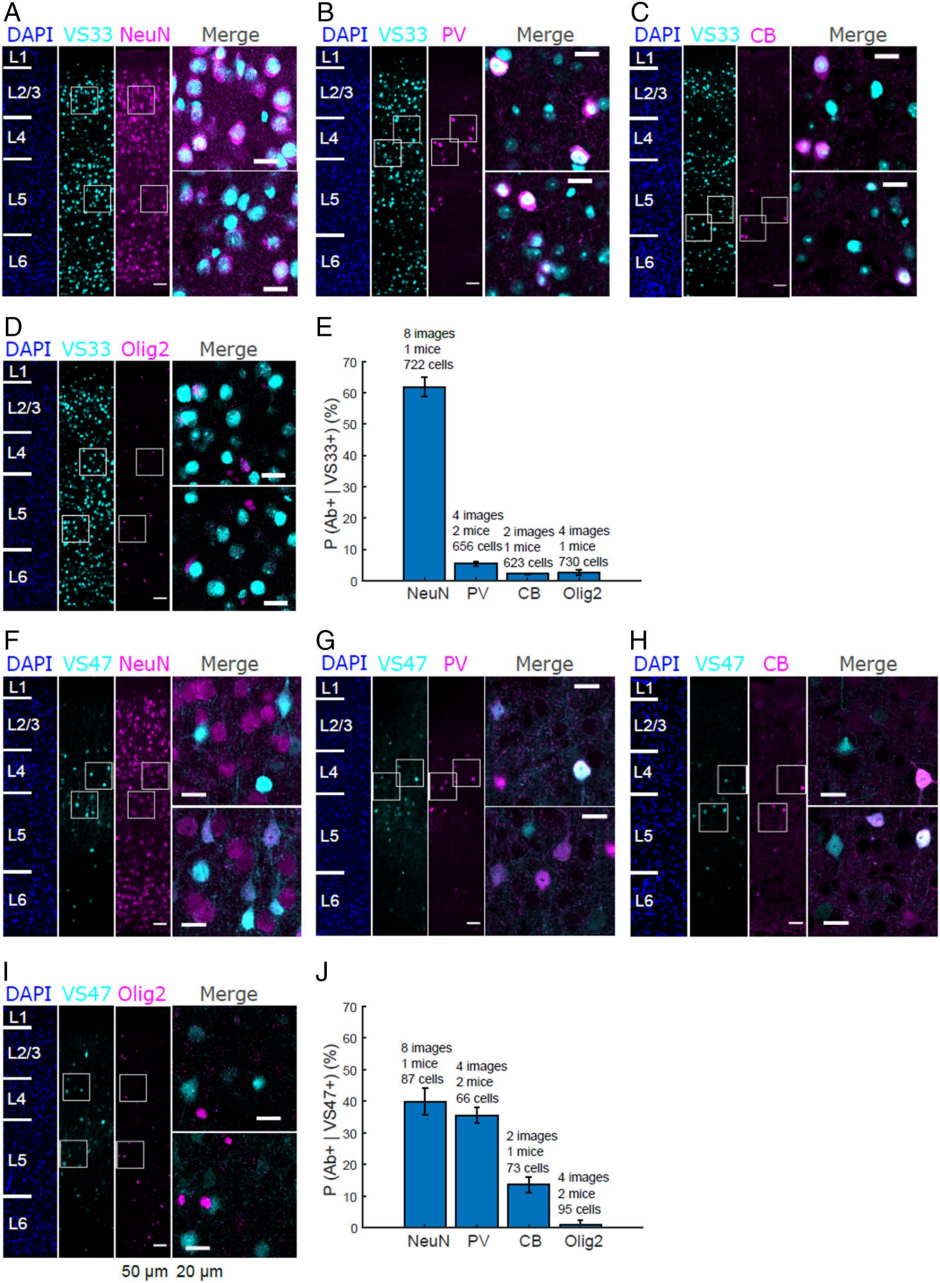
Cell type characterization of validation sequences VS33 and VS47 using immunostaining in coronal sections of the auditory cortex. (**A**–**D**) Fluorescence microscopy images of VS33-driven mTFP expression (cyan) and immunostaining of cell type marker antibodies (magenta) in the same fields of view. Cell type marker proteins are NeuN (A), Parvalbumin (PV) (**B**), Calbindin (CB) (**C**) and Olig2 (**D**). Six neocortical layers indicated on DAPI image. (**E**) Quantification of the percentage of VS33-positive cells that are co-stained by the cell type markers in (A-D) (mean across images ± SD). VS33 drives expression in mainly neuronal cells. (**F**–**J**) Analogous to (A-E), but for VS47-driven mTFP expression. VS47 drives expression prominently in the interneurons.

To further characterize the types of cells in which these candidate enhancers are active, we performed immunostaining of cell type marker proteins which allowed us to obtain data from a larger number of cells as compared to semi-automated reconstructions of individual cells. We injected mouse brains with the reporter constructs driving nuclear-localized mTFP under the control of the candidate enhancers. After three weeks, we sacrificed the mice and immunostained cortical slices with antibodies against several biomarker proteins to label neurons (NeuN), parvalbumin positive interneurons (Parv), calbidin positive interneurons (CB), and oligodendrocytes (Olig2) respectively. We then quantified the co-labeling of the antibody stains with the fluorescence signal from the injected VS33 and VS47 mTFP reporters in confocal images (Fig. 6). For VS33, we observed broad expression in neurons, but rarely overlap with parvalbumin + or calbindin + interneurons or with oligodendroctyes (Fig. 6A–E; 62%, 4%, 2% and 4% of VS33 + cells co-stained NeuN + , PV + , CB + , and Olig2 + respectively). VS47, on the other hand, drives expression in neurons as well as in parvalbumin + interneurons, but sparsely in calbindin + interneurons and scarcely in oligodendroctyes (Fig. 6F–J; 40%, 28%, 14% and 2% of VS47 + cells co-stained NeuN + , PV + , CB + , and Olig2 + respectively). Therefore, both candidates drive expression in neurons, but with different subtype preference. Neither candidate showed significant activity in oligodendrocytes.

**Figure 7:**
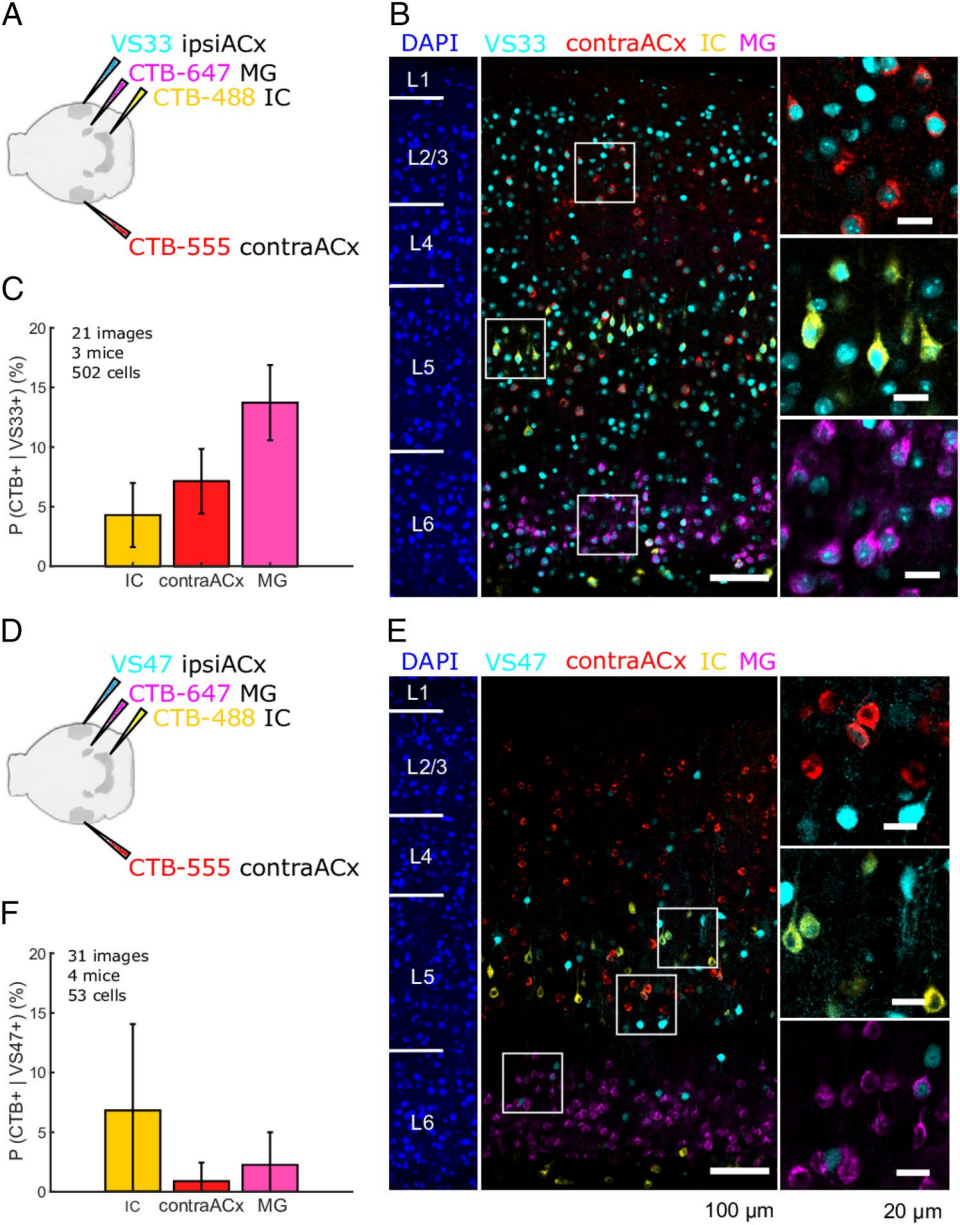
Projection patterns of neurons accessed with VS33 and VS47. (**A**) Schematic showing locations of the AAV8 capsid injections and the fluorescence colors for the VS33-driven mTFP reporter (cyan) and the CTB-conjugated fluorophores for retrograde labeling (red, yellow and magenta). (**B**) Multicolor fluorescence image of auditory cortex injected with AAV8 capsids containing VS33-driven mTFP (cyan), along with retrograde-labeled neurons projecting to the contralateral auditory cortex (red), the inferior colliculus (yellow) and the medial geniculate nucleus (magenta). Right panels are expansions of the insets. (**C**) Quantification of the percentage of VS33-positive cells that are co-stained by the CTB-conjugated fluorophores (mean across images ± SD). VS33 drives expression in projecting cortical cells which predominantly project to MG. (**D**–**F**) Analogous to (**A**–**C**), but for the VS47-driven mTFP reporter construct. VS47 drives expression in projecting cortical cells which predominantly project to IC. IpsiACx: Ipsilateral auditory cortex; contraACx: contralateral auditory cortex; IC: inferior colliculus; MG: medial geniculate nucleus.

Finally, we investigated the projection patterns of neurons in which these candidate enhancers are active using cholera toxin B subunit (CTB) for retrograde tracing (Fig. 7). CTB is taken up by axon terminals, travels retrogradely and leads to an intense labeling of the cell bodies of the projection neurons when conjugated with an Alexa fluorophore. To analyze the projection preferences of neurons in which the VS33 and VS47 sequences are active, we injected the reporter constructs driving nuclear-localized mTFP under the control of the candidate enhancers in the auditory cortex. In addition, we performed stereotaxic injections in three major projection targets of auditory cortex projection neurons, namely the contralateral auditory cortex (ACx), the ipsilateral medial geniculate nucleus (MG) and the ipsilateral inferior colliculus (IC) using three different colored CTB-conjugates. After three weeks of incubation time, we performed multi-channel fluorescence confocal microscopy in coronal sections of the auditory cortex and other injections sites to confirm successful targeting of projection areas. Consistent with previous reports^40^, neuronal somata retrogradely labeled from the contralateral auditory cortex were primarily found in layers 2–5, neuronal somata retrogradely labeled from the inferior colliculus were found primarily in layer 5 and neuronal somata retrogradely labeled from the medial geniculate nucleus were primarily found in layer 6. When quantifying the co-labeling of the three retrograde labels with the reporter signals driven from VS33, we found a bias towards neurons projecting to the MG as compared to contralateral ACx and the lowest preference for IC-projecting neurons (Fig. 7A–C; 4.4% of IC + , 7.2% of contraACx + , and 13.7% of MG + cells were also VS33 +). In contrast, VS47 showed in general lower levels of co-labeling with the retrograde labels, consistent with an expression bias towards classical non-projecting neurons. Here, the preference was towards IC-projecting neurons (Fig. 4D–F; 8.5% of IC + , 1.1% of contraACx + , and 2.8% of MG + cells were also VS47 +).

In summary, our histological analysis allowed us to validate several genomic elements driving differential expression patterns also in sparse cell types that have been identified in an unbiased AAV-STARR-seq screen.

## Discussion

Here, we report the results of an unbiased AAV-STARR-seq screen of entire genomic regions in mouse brains to obtain functional maps of enhancer activities in vivo. By establishing a complex screening library and applying efficient delivery to the mouse brain, we were able to screen hundreds of thousands of different RNA sequences in an individual mouse (Supplemental Table 2), which would have been challenging using traditional MPRA assays involving barcodes. This substantial increased in scaling allowed us to no longer assess enhancer activities of a limited number of selected candidate enhancers, but to tile genomic regions in an unbiased manner in order to create entire activity maps. The nine genomic regions spanned in total about 3 Mb, which comprises about 0.1% of the full mouse genome. The remarkably high reproducibility of the RNA signal between mice indicates that the targeted regions are saturated with RNA coverage and therefore could be scaled up to a larger subset of the mouse genome. Scaling AAV-STARR-seq up to the full genome scale in individual mice, as previously demonstrated in human cell lines^28–30^, may be nevertheless challenging given the limited quantity of AAV that can be administered. Most importantly, while still focusing on genomic loci of target genes, the AAV-STARR-seq approach allowed us to overcome the necessity to pre-select specific candidate sequences that are typically nominated based on accessible chromatin regions or enhancer-associated histone marks.

In fact, the majority of sequences identified in the AAV-STARR-seq screen were not overlapping with chromatin marks listed in ENCODE databases. Several factors can contribute to this finding. On the one hand ChIP signals stemming from rare cell types may not have been strong enough to be included. On the other hand, some genomic sequences may show enhancer activity in brain cells unless they are silenced by chromatin modifications. After transduction AAV genomes remain essentially episomal and lacks chromatin.

The strongest enhancer candidate we identified, VS33, showed a clear and strong signal in both the STARR-seq screeen and the fluorescence reporter validation, where we observed a broad expression pattern in neurons across cortical layers. Interestingly, this enhancer is located at the promoter of the Lin7a (Lin-7 homolog A, crumbs cell polarity complex component) gene spanning its transcription start sites (Supplemental Fig. S3A). Such promoters that can also act as distal enhancers are relatively rare, constituting just 2–3% of promoters in human HeLa-S3 and K562 cell lines^27,41^, highlighting that enhancer screens should ideally not be based on prior assumptions about potentially suitable candidates. The Lin7a gene itself is a broadly expressed but brain-enriched gene involved in coupling synaptic vesicle exocytosis to cell adhesion in the brain^42,43^, consistent with the neuronal preference of VS33-driven expression.

The other enhancer we characterized in detail, VS47, was not associated with open chromatin or active enhancer-associated histone marks in the adult mouse brain, such as the majority of the STARR-seq peaks obtained in our screen. In the histological validation, VS47 drove an expression pattern in a cortical layer-specific manner that also showed a cell type-specificity and regional connectivity pattern that was distinct from the VS33 candidate enhancer. It is important to note that we would not have identified this enhancer if we had pre-selected our enhancer candidates based on epigenetic data before screening. A potential challenge to our approach to use totalRNA isolated from a block of tissue comprised of a multitude of cell types, is that signals from very rare cell types may fall below the detection threshold. However, our finding that VS47 drove expression only in a very sparse subset of neurons, indicates that the AAV-STARR-seq screen has a remarkably high sensitivity to detect signals that stem from only a minor fraction of cells.

Applying unbiased STARR-seq screens to living animals significantly widens the possibilities to directly functionally assess enhancer activities in cells that are embedded in their physiological environment. This is a crucial difference to functional screens that are typically performed in cell lines based on immortalized or tumor cells. Available culture systems for post-mitotic, fully differentiated somatic cells, as they are particularly relevant for the brain, are limited. Furthermore, assessing enhancer activity in vivo is particularly important for the study of state-dependent gene regulation, where environmental interactions can influence gene expression. For example, it has been shown that learning and memory formation induces remodeling of the neuronal chromatin landscape at the microscopic^44^ and molecular level^45^.

Apart from the study of the biological function of regulatory elements, AAV-STARR-seq can be used to identify enhancers with interesting properties to construct small, artificial promoter elements for viral vectors by combining an enhancer with a minimal core promoter^8–14^. AAV-STARR-seq represents a particularly useful complement to identify sequences that work as enhancers in a context that is free from chromatin, such as AAV-based vectors that typically remain episomal. Here, the predictive power of approaches for enhancer identification based on epigenetic marks is limited.

AAV have become a widely-used vector combining efficient gene delivery with a high safety profile^46,47^. The development of small promoter elements for cell-type specific gene delivery is as important for basic research as for the clinical context. In medicine, AAV-based gene therapy has become a clinical reality and first FDA-approved products like Luxturna or Zolgesma bring novel therapies to patients with so far untreatable disorders^48,49^. Naturally occurring AAV capsid serotypes and engineered variants of these wildtype capsids are currently expanding the toolbox of researches to target various organs or cell types in different mammalian species^7,48^. However, the tropism of most available capsids is often not specific to one single cell type or tissue^32^. This necessitates the implementation of cell-type specific enhancers and promoters into the AAV expression cassette^50,51^ to further increase the specificity of therapeutic gene expression. Therefore, the identification of suitable novel regulatory elements is also of high interest for the entire field of AAV based gene therapy^48^.

## Methods

### Cell culture

HEK293 cells were purchased from American Type Culture Collection (ATCC; cat. no. CRL-1573). Cells were cultured in DMEM (Gibco; cat. no. 52100-047), supplemented with 10% heat-inactivated FBS (Sigma; cat. no. F7524), 2 mM L-glutamine (Sigma; cat. no. G7513) and 1% Penicillin–Streptomycin (Sigma-Aldrich cat. no. P0781-100ML) in a carbon dioxide (CO_2_) incubator (37 °C temperature, 95% relative humidity and 5% CO_2_). Cells were harvested at 80% confluence by removing the growth medium, washing with 1 × PBS, treating with 0.25% Trypsin–EDTA (Gibco; cat. no. 25200–056) until dispersion of the cell layer and resuspension in complete medium.

### BACs: Propagation and purification

Bacterial artificial chromosomes (BAC) were purchased from BACPAC Genomics Inc. (Richmond, California, USA). BAC clone IDs, sizes and mouse chromosomal regions, which they cover are listed in Supplemental Table 1 (https://bacpacresources.org/home.htm). BACs were provided as LB agar stab cultures (DH10B *E. coli* host) and streaked to single colonies on LB agar plates supplemented with 12.5 µg/ml chloramphenicol. Individual colonies from each BAC were further propagated in 500 ml LB media with appropriate antibiotic. BACs were purified using the QIAGEN large construct kit (cat. no. 12462) according to the manufacturers protocol. The protocol included an ATP-Dependent Exonuclease digestion step to ensure selective removal of contaminating genomic DNA, as well as nicked or damaged BAC DNA.

### BACs: STARR-seq library preparation

The STARR-seq screening library was prepared as previously described. For a detailed protocol, please refer to http://starklab.org/data/muerdter_boryn_2017/. In brief, BACs listed in Supplemental Table 1 were mixed at equimolar ratio to a total of 30 µg. Total volume was adjusted to 600 µl with ddH_2_O. Fragmentation was performed with a Covaris S2 ultrasonicator in Micro-Tube-50 AFA-fibre-screw-cap tubes with a sample volume of 50 µl per reaction. Target peak size of above 500 bp (> 500) was achieved with the following settings: Intensity level 5; 5% duty cycle; 200 cycles per burst; 40 s duration, at 7 °C and a water level of 15.

Six reactions were performed in parallel. Samples were pooled again afterwards and loaded on a 1.2% agarose gel with Ethidium Bromide staining. Gel electrophorese was performed at 90 Volt (constant) for 30 min. BAC fragments with a size between 500 and 1000 bp were cut from the gel and purified with a commercially available kit (Qiagen; Cat. No. 28706X4) according to the manufacturer’s protocol. Average fragment size was determined to be 742 bp with a Bioanalyzer. Library integrity and purity was confirmed by MiSeq NGS.

We performed four parallel ligations of hairpin-adapters (NEB; Kit: E7335) for the library generation. Adapter ligation was performed according to the protocol described in the manual NEBNext Ultra II DNA Library Prep Kit for Illumina (NEB; E7645). The adapter ligated DNA library was purified twice with AMPure XP beats (Beckman Coulter; Cat. No. A63881) according to the original protocol. Second purification step was performed to ensure removal of adaptor dimers.

For PCR amplification of the adapter-ligated DNA library we used the KAPA HiFi HotStart Ready Mix (2x) (KAPA; Cat. No. KK2601) according to the manufacturers protocol with the following primers. In-Fusion_fwd: 5’-*TAGAGCATGCACCGG*ACACTCTTTCCCTACACGACGCTCTTCCGATCT-3’ and In-Fusion_rev: 5’- *GGCCGAATTCGTCGA*GTGACTGGAGTTCAGACGTGTGCTCTTCCGATCT-3’. Underlined, italic parts of the oligos are homologous to the sequences of the AAV screening vector (described below) flanking the library insertion site. These homology arms are necessary for InFusion cloning. The parts of the homology arms shown in bold are truncated recognition sites for restriction enzymes AgeI and SalI, respectively. Therewith it is ensured that the respective restriction sites are no longer present in the screening vector once it has received a library fragment. Eight PCR reactions were performed in parallel with 1 µl of purified adapter ligated DNA per reaction and eight PCR cycles. Samples were pooled afterwards and integrity of the PCR product was confirmed by gel electrophoresis of a 3 µl aliquot on a 1.2% agarose gel with Eth Br staining. The remaining PCR product was purified AMPure XP beats and subsequently with the QIAquick PCR Purification Kit (Qiagen; Cat. No. 74104). The subsequent purification was found critical for a high efficiency in the downstream InFusion cloning reactions.

The AAV screening vector (described below) was digested with AgeI-HF and SalI-HF, respectively (NEB; Cat. No. R3552; R3138). 10 µg of plasmid was mixed with 5 µl AgeI-HF (100 units), 5 µl SalI-HF (100 units), 15 µl 10 × Cut Smart buffer (provided with the enzymes) and dd H2O was added to a total of 150 µl. The reaction was incubated at 37 °C for 4 h and heat inactivated at 65 °C for 20 min. Digested plasmid DNA was purified by agarose electrophoresis on a 1% agarose gel with Eth. Br. staining. Plasmid DNA band was cut from the gel and purified with a commercially available kit (PeqLab X) according to the manufacturer’s protocol. Subsequent purification steps with the OIAquick PCR purification kit (Qiagen; Cat. No. 74104) and the MinElute PCR Purification Kit (Qiagen; Cat. No. 28006) were performed according to the original protocol (Muerdter et al., 2017). As for the PCR amplified BAC DNA library a high purity of the digested vector DNA was found critical for efficient InFusion cloning.

To clone our PCR amplified BAC DNA library into the AAV-STARRseq vector we used the InFusion HD cloning Kit (Clontech; Cat. No. 639650). Twelve reactions instead of four were performed as previously described. Precipitation of the ligated DNA library previous to electroporation is critical for efficient transformation. Four ligation reactions were pooled for one precipitation reaction. Three precipitation reactions were done in parallel and pooled afterwards to a total of 37.5 µl. Please note that the total amount of precipitated DNA exceeds the amount needed in the downstream transformation.

Transformation was done by electroporation of MegaX DH10B electrocompetent E. coli. (Invitrogen; Cat. No. C640003). Ten transformations with ligated BAC DNA library plus one pUC19 control (provided with the bacteria) were prepared by mixing 2.5 µl BAC library DNA or 1 µl pUC19, respectively with 20 µl MegaX DH10B. Electroporation was done in Gene Pulser Electroporation Cuvettes with 0.1 cm cap (Biorad; Cat. No.1652089) on a GenePulserxCell Electroporator (Biorad). Settings were as follows: Constant protocol, 2 kV, 25µF and 200 ohms. Bacteria were recovered immediately after electroporation in 1 ml recovery media (provided with the bacteria) per transformation, at 37 °C in an incubator shaking at 300 rpm. After recovery samples were pooled. A dilution series was done from 100 µl bacterial culture and streaked on LB agar plates with 100 µg/ml Ampicillin and incubated o.n. at 37 °C, to determine the efficiency of the transformation. The remaining bacterial culture was equally distributed to 6 × 1L LB media supplemented with 100 µg/ml Ampicillin, in 2L Erlenmeyer flasks and incubated o.n. for 13 h to 16 h at 37 °C, shaking at 200 rpm. OD of the bacterial culture was determined the next day. At an OD of 2–2.6 bacteria were harvested by centrifugation for 30–45 min at 4200 rpm at 4 °C. Bacterial pellets were resuspended in 10 ml LB media each and pool. The pooled bacterial suspension was distributed equally to five 50 ml tubes and centrifuged 15 min at 6000 × g at 4 °C. After aspiration of the supernatant bacterial pellets were kept at −20 °C. Cloned BAC DNA plasmid library was purified from one pellet with a Plasmid Gigaprep Kit (Qiagen; cat. no. 12191) according to the manufacturer’s protocol.

### AAV-STARR-seq screening vector

The AAV-STARR-seq screening plasmid is based on the STARR-seq screening vector previously described by Arnold and colleagues (Addgene #71509)^27^. The plasmid was digested with PspOMI and AfeI and size selected by gel electrophoreses on an 1% agarose gel with Eth. Br. staining. The entire cassette, spanning from the chimeric intron to the SV40 late polyA signal, including the truncated GFP (tGFP), Canamycin resistance and ccdB survival cassette was purified and transferred to a pAAV recipient plasmid. The insertion site in pAAV is flanked by inverted terminal repeats. A minimal promoter, derived from plasmid GL4.26 (Promega Cat. No. E8441), was inserted between 5’ ITR and tGFP. Sequence integrity was confirmed by sequencing. Integrity of the ITRs was confirmed by restriction digest with SrfI. Please note that the terminal resolution site of the 3’ ITR is mutated, which leads to a concatemerization of the positive and negative strand during AAV transgene replication. As a result, each viral capsid packages a pair of complementary sequences of the transgene.

### AAV-STARR-seq validation vectors

The plasmids for multiplexed validation are based on pBS(SK-) that contained a CMV promoter, a ß-Globin intron, a WPRE site and a BGH polyA site. The entire expression cassette is flanked by ITR’s from AAV2. In addition, three nuclear localization signals fused to either one of the following fluorophore genes: mTFP, eGFP, Venus or tagRFP were cloned downstream of the ß-Globin intron. To generate the CMV driven normalization control, a 1 kb stuffer sequence was cloned via MluI sites upstream of the CMV promoter in each of these plasmids. The stuffer consists of a sequence (Seq17) that was found inert in previous STARRseq screens. To generate the constructs containing the validation sequence, the CMV promoter together with the stuffer sequence were cut out with MluI and AgeI restriction enzymes and replaced by a respective validation sequence followed by the 4.26 minimal promoter. Validation sequences differ in size between 1.2 and 1.5 kb. Depending on the size of the VS, stuffer sequences of different length from Sequ17 were cloned between 5’ITR and VS. The stuffer sequences in both the normalization control and the VS constructs serve two purposes: First, to keep the size of the entire cassette between the ITRs at constant 4.5 kb and second to hamper potential promoter activity of the 5’ITR.

Plasmids for single construct validation were produced by Vector Builder according to our specifications. They contain VS33 or VS47, respectively, upstream of the 4.26 minimal promoter and the ß-Globin intron. The iCre gene separated by a P2A site from a TFP with 3NLS; is located downstream, followed by a WPRE site and a BHG polyA site. The entire cassette is flanked by ITR’s from AAV2.

### AAV production

For AAV production 3 × 10^7^ HEK 293 cells were seeded in a 16-layer Celldisc (Greiner; Cat. no. 678916) with 1L complete growth media (DMEM, Gibco; Cat. No. 52100–047), supplemented with 10% heat-inactivated FBS (Sigma; Cat. No. F7524), 2 mM L-glutamine (Sigma; cat. no. G7513) and 1% Penicillin–Streptomycin (Sigma-Aldrich Cat. No. P0781-100ML) and cultured for 48 h in CO_2_ incubator (37 °C temperature, 95% relative humidity and 5% CO_2_)^52^. For chemical transfection plasmid pADDeltaF6 (Addgene Cat. No. #112867), pAAV8 (Addgene Cat. No. # 112864) and BAC-DNA plasmid library were mixed at equimolar ratio to a total of 2.069 mg DNA. 69 ml of 300 mM CaCl2 was added to the plasmid DNA. The entire CaCl2/DNA mixture was slowly added to 69 ml 2xHBS solution (Aesar; Cat. No. #J62623). After 5 min. incubation the mixture was added to 500 ml DEMEM supplemented with 5% FCS (no antibiotics). Culture media was then carefully decanted from the Celldisc and replaced with the transfection media. After 6 h incubation (37 °C, 95% relative humidity and 5% CO_2_) transfection media was carefully decanted and replaced with 1L of fresh complete growth media. Transfected cells were incubated for 72 h (37 °C, 95% relative humidity and 5% CO_2_).

To harvest the cells growth media was carefully decanted and collected. 500 ml of kept growth media was supplemented with 7 ml 0.5 M EDTA (Invitrogen; Cat. No. #15575-020) and 400 ml out of it was put back into the Celldisc. After 5 min incubation at room temperature cells detached from the surface. Cell suspension was transferred to a 500 ml centrifugation flask (Corning; Cat. No. 431123). The remaining 100 ml Growth media/EDTA mix was used to wash the Celldisc and added to the centrifugation flask.

After centrifugation at 800 × g for 15 min at 4 °C, supernatant was carefully discarded. The cell pellet was resuspended in 10 ml PBS, transferred to a 50 ml Falcon tube and centrifuged again for 15 min at 800 × g, at 4 °C. PBS was then discarded and the pellet resuspended in 24 ml lysis buffer (50 mM Tris, 1 M NaCl, 10 mM MgCL2) supplemented with 0,001% Pluronic F-68: (Invitrogen #24040032), 1300U Salt Active Nuclease (SAN) (Sarstedt #83.1803) and 100 × HALT Protease Inhibitor Cocktail, (EDTA-free Thermo scientific #78439). Cell suspension was then subjected to three freeze / thaw cycles in liquid nitrogen and a 37 °C water bath, respectively. To assure that the suspension does not contain any remaining plasmid DNA, it was again supplemented with 1300U of SAN afterwards, and incubated at 37 °C for 1 h, while shaking at 150 rpm. Following centrifugation at 2500 × g for 15 min at r.t, cell debris was discarded, and the supernatant was transferred to a new 50 ml Falcon tube. 40% PEG-8000 solution (Polyethyleneglycol, Sigma #89510, in H2O, supplemented with 0.001% Pluronic) was added to a final concentration of 8%, mixed and incubated on ice at 4 °C for 16 h to 24 h. After centrifugation at 2500 × g for 30 min at 4 °C, supernatant was discarded, any residual PEG was carefully removed and 14.5 ml resuspension buffer (50 mM TRIS, 1 M NaCl, 0.001% Pluronic, pH8.0) was added to the pellet and the pellet was resuspended by vortexing and pipetting before it was incubated for at least 24 h at 4 °C, while shaking at 350–400 rpm. It was found crucial to resuspend the pellet completely. The suspension was then centrifuged at 2500 × g for 30 min at 4 °C and the supernatant was transferred to an ultra-centrifugation tube (Quickseal Tubes, Beckman Coulter #342414). AAV purification was performed by ultra-centrifugation over a discontinuous Iodixanol density gradient (OptiPrep Density Gradient Medium, Sigma #D-1556, 60% solution in H2O), as described previously^53^ with Iodixanol phases of 15%, 25%, 40% and 54%, respectively. After centrifugation, approximately 3.5 ml of the Iodixanol phases containing the filled AAV capsids were collected (2.5 ml of 40% and 1 ml of 54% phase). Special care was taken not to touch the 25% phase, since it contains empty capsids. For buffer exchange and concentration, AAV purification buffer (1 × PBS, 1 mM MgCl2, 2.5 mM KCL, 0.001% Pluronic, pH 7.4) was added to the virus containing fraction, to a total volume of 12 ml and transferred to a 15 ml AMICON ULTRA-15 column; (MWCO 100 kDa, Millipore #UFC910024). Centrifugation was performed according to the manufacturers protocol. After concentration of the virus solution to approximately 1.5 ml, fresh AAV purification buffer was added to a total volume of 12 ml, and centrifugation was repeated. At a volume of 1 ml to 1.5 ml, virus solution was resuspended thoroughly, transferred to a new tube and stored at -80 °C. The genomic titer was determined by qRT-PCR.

### AAV genomic titer determination by qRT-PCR

The genomic titers of our AAV productions were determined by qRT-PCR on an ABI qRT thermal cycler with primer combinations and FAM/TAMRA labeled probes, specific to the respective target sequences. Titers of STARR-seq screening vectors were determined with primers and probes that bind within the chimeric intron and the truncated GFP, respectively (pAAV_BAClib_qRT_forw: 5’- GAGACAGAGAAGACTCTTG -3’; pAAV_BAClib_qRT_rev: 5’- GCTCTAGATCAATCTAATTCAA -3’; pAAV_Val_seq_qRT_rev: 5’- GTGGACACCTGTGGAGAGA -3’; pAAV_BAClib_qRT_probe: FAM-5’- AGGCACCTATTGGTCTTACTGACATC -3’TAMRA). Titers of the STARR-seq validation vectors were determined using primers / probe combinations that bind within CMV or the respective fluorophore sequence. Primers / probes for GFP were also suitable for Venus detection. (CMV_qRT_forw: 5’- GTGGGAGGTCTATATAAGC -3’; CMV_qRT_rev: 5’- GTGTCTTCTATGGAGGTC -3’; CMV_qRT_probe: FAM-5’- AGTGAACCGTCAGATCGCCT -3’-TAMRA GFP_qRT_forw: 5’- GCTGGAGTACAACTACAAC -3’; GFP_qRT_rev: 5’- TGGCGGATCTTGAAGTT -3’; GFP_qRT_probe: FAM-5’- CTTGATGCCGTTCTTCTGCTTGT -3’-TAMRA; BFP_qRT_forw: 5’- ACCACATATAGATCCAAGA -3’; BFP_qRT_rev: 5’- GTCTGTAGTCCACATAGTA -3’; BFP_qRT_probe: FAM-5’- CGCTAAGAACCTCAAGATGCCTG -3’-TAMRA; RFP_qRT_forw: 5’- GACCACATACAGATCCAA -3’; RFP_qRT_rev: 5’- CCTCCTTGATTCTTTCCA -3’; RFP_qRT_probe: FAM-5’- CCGCTAAGAACCTCAAGATGCC -3’-TAMRA).

Titers of AAV samples were determined by comparison to a standard curve compiled from a serial dilution of plasmid standards of known concentration, ranging from 5E + 4 to 5E + 8 copies per reaction. For each primer/ probe combination a separate standard curve was generated. Plasmids used as standards are the same as for AAV production.

Sample preparation was done according to the following protocol: Viral ssDNA was isolated from 2 µl of purified AAV, mixed with 50 µl ABI buffer (100 mM KCL; 20 mM Tris pH 7.4; 10 mM MgCl2), 46 µl nuclease free H2O and 1 µl DNase TypeIV (Roche). Reaction mixture was incubated for 30 min at 37 °C followed by 10 min at 75 °C to inactivate DNase. 1 µl (10 µg) Proteinase K was added to the mixture and incubated for 1 h at 50 °C before the reaction was stopped at 95 °C for 20 min. The reaction mixture was then further diluted 1:25 with nuclease free water. Reaction mixture for qRT-PCR was set up as follows: 400 nM of each forward and reverse primer, 100 nM probe labelled with 6FAM/TAMRA, 1.4 µl of diluted sample or plasmid standard, respectively, and 5 µl of Fast Advance Mastermix (ABI) were mixed on ice in a 10 µl reaction. Cycling conditions on the ABI qRT thermal cycler were set to an initial 2 min at 50 °C and 20 s at 95 °C, followed by 40 cycles of 1 s at 95 °C and 20 s at 60 °C. Data analysis was performed using the ABI software.

### Animals

Male laboratory mice (C57BL/6JRj) were purchased from Janvier laboratories and kept in groups of up to five in 530 cm^2^ cages on a 12 h light/dark cycle with unlimited access to dry food and water. Mice were aged between 6 and 10 weeks of age when entering the experiments. All animal experiments were approved by the Landesuntersuchungsamt Rheinland Pfalz (Approval 23 177–07/G 17–1-052) and followed European guidelines for animal research. The methods and results are reported in accordance with ARRIVE guidelines.

### Stereotaxic injection

Mice were deeply anesthetized before surgery and the parietal bones of the skull exposed, cleaned and small holes were drilled with a stereotactic drill. BAC library (1.52 × 10^12^ vg/ml) was injected (200 nl per site for twelve sites) in somatosensory, motor, retrosplenial and parietal association areas of the neocortex (0.5–2.5 mm posterior, 1–2 mm lateral, relative to bregma, and 0.6 mm ventral relative to the brain surface). VS construct (multiplexed fluorescence construct: 3.4 × 10^12^ vg/ml; VS33: 1.14 × 10^12^ vg/ml; VS47: 3.16 × 10^12^ vg/ml) was injected (150–200 nl per site for three sites) in the auditory cortex (4.2–4.6 mm lateral, 3 mm posterior, and 2.5–2.7 mm ventral relative to bregma). For CTB retrograde labeling, 1% CTB was injected in the inferior colliculus (500 nl of CTB-alexa fluor 488; 0.6–1.5 mm lateral, 5 mm posterior, 1.5 mm ventral relative to bregma), the contralateral auditory cortex (500 nl of CTB-alexa fluor555; 4.4 mm lateral, 3 mm posterior, 2.6 mm ventral relative to bregma), and the medial geniculate nucleus (150 nl of CTB-alexa fluor 647; 2.3 mm lateral, 3 mm posterior, 3.3 mm ventral relative to bregma). For sparse whole cell labeling, VS construct was diluted (1:5000) and co-injected with EF1a-DIO-EYFP (Addgene, 27,056-AAV9) at a 1/1 ratio. Injection was administered at the rate of 20 nl/min and the glass pipette was left in place for 5 min after the injection to minimize the backflow. After injection, the skin was closed with Vetbond and mice were relieved from medetomidine with atipamezole.

### Sample preparation and next-generation sequencing

For sample preparation and sequencing, we followed the general recommendations for STARR-seq as outlined in^30^.

*Preparation of AAV-STARR-seq samples*: One week after injection, six injected mice were sacrificed by cervical dislocation. The cortical tissue surrounding the injection sites was quickly removed and frozen using liquid nitrogen. Total mRNA was isolated from individual brain tissue samples using the AllPrep DNA/RNA Mini Kit (Qiagen; Cat. No. 80204) according to the manufacturer’s protocol (version 2005). In brief, frozen tissue samples were weighed not to exceed 30 mg per sample. The maximum recommended volume of 600 µl RLT Plus buffer, supplemented with 10 μl β-ME per 1 ml Buffer RLT Plus, was added to the frozen tissue. Samples were then passed 25 times through a 20-gauge needle fitted to an RNase-free syringe. Thorough homogenization was found crucial for good RNA and DNA yield and quality. After centrifugation, the supernatant was carefully aspirated and transferred to an DNA binding column. Isolated DNA was kept for internal quality control. The flow through was collected and transferred to RNA binding columns, centrifuged and washed. An on-column DNase Digestion was performed using the recommended RNase-Free DNase Set (Qiagen #79,254), following the steps as described in Appendix E of the AllPrep DNA/RNA Mini Kit protocol. After 15 min at r.t. columns were washed again, and total RNA was eluted in 30 µl RNAse free water.

Oligo-dT selection of mRNA was performed with NEBNext® Poly(A) mRNA Magnetic Isolation Module (NEB; Cat. No. E7490S/L) with a starting amount of 5 µg (except for sample 1 with a starting amount of 2.7 µg). Reverse Transcription was performed with SuperScriptIII (Invitrogen; Cat. No. 18080093) using a Gene specific primer (CTCATCAATGTATCTTATCATGTCTG). RT was followed by RNAse Treatment and junction PCR with 15 cycles. Libraries were amplified in 8 Cycles. Libraries were profiled in a High Sensitivity DNA on a 2100 Bioanalyzer (Agilent technologies) and quantified using the Qubit dsDNA HS Assay Kit, in a Qubit 2.0 Fluorometer (Life technologies).

*Preparation of viral DNA as input control*: Three replicate samples of viral DNA were obtained from the packaged screening library using the Qiagen MinElute Virus Spin Kit (#57,704) following the protocol for Purification of Viral Nucleic Acids from Plasma or Serum. In brief, 25 µl of purified AAV was supplemented with 175 µl 0.9% sodium chloride, to a total volume of 200 µl and added to 25 µl QIAGEN protease (provided with the kit). 200 µl of AL buffer was added to the mixture. It is important to note that the AL buffer did not contain any carrier RNA, since the RNA would interfere with downstream applications. After incubation for 15 min at 56 °C, 250 µl of 100% Ethanol was added and after another 5 min of incubation at room temperature the entire mixture was applied to a QIAamp MinElute column to bind viral DNA. Following centrifugation, flowthrough was discarded, and the column was washed with 500 µl AW1 and AW2 respectively. After a final washing step with 500 µl of 100% ethanol the column was centrifuged at full speed for 3 min and subsequently dried for another 3 min at 56 °C to ensure the membrane does not contain any residual ethanol. To elute the viral DNA, 30 µl of RNAse/ DNase free water was applied directly to the membrane, incubated for 2 min at room temperature and centrifuged at full speed for 1 min. Sequencing libraries of viral DNA were prepared with a starting amount of 10 ng and were amplified in 12 PCR cycles using the KAPA HotStart ReadyMix (Kapa Biosystems). Libraries were profiled in a High Sensitivity DNA on a 2100 Bioanalyzer (Agilent technologies) and quantified using the Qubit dsDNA HS Assay Kit, in a Qubit 2.0 Fluorometer (Life technologies).

*Sequencing*: All six AAV-STARR-seq samples were pooled in equimolar ratio together with the 3 viral DNA libraries and sequenced on a NextSeq 500/550 Flowcell, PE for 2 × 79 cycles plus 7 cycles for the index read resulting in about 9–19 million reads per sample, with an average of 13.6 million. Sample 6 was excluded from further analysis as it had substantially fewer sequenced reads than the other samples.

### Next-generation sequencing data availability

The data has been submitted to the Gene Expression Omnibus (GEO) under the accession GSE197005.

Furthermore, a public UCSC Genome Browser session was created to view the tracks associated with this manuscript.

It can be activated by loading the following URL into a web browser:https://genome-euro.ucsc.edu/s/moti/Chan2022_BACs_AAV_STARRseq.

Once activated, the nine genomic regions assayed in this study can be viewed at these URLs:https://genome-euro.ucsc.edu/cgi-bin/hgTracks?db=mm10&position=chr10:107113919-107310064.

https://genome-euro.ucsc.edu/cgi-bin/hgTracks?db=mm10&position=chr11:102829076-103027447.

https://genome-euro.ucsc.edu/cgi-bin/hgTracks?db=mm10&position=chr12:38595495-39036494.

https://genome-euro.ucsc.edu/cgi-bin/hgTracks?db=mm10&position=chr16:23798011-24077296.

https://genome-euro.ucsc.edu/cgi-bin/hgTracks?db=mm10&position=chr18:60715061-61136779.

https://genome-euro.ucsc.edu/cgi-bin/hgTracks?db=mm10&position=chr2:22551742-22920596.

https://genome-euro.ucsc.edu/cgi-bin/hgTracks?db=mm10&position=chr5:36741605-37215239.

https://genome-euro.ucsc.edu/cgi-bin/hgTracks?db=mm10&position=chr6:85116745-85346354.

https://genome-euro.ucsc.edu/cgi-bin/hgTracks?db=mm10&position=chrX:20911236-21111402.

### Next-generation sequencing data processing

The study design included only a single experimental group, therefore no blinding or randomization procedures were applied. Quality control of sequenced reads was performed using FastQC (v. 0.11.8). Sequencing reads were mapped to the mouse mm10 genome assembly using bowtie2 (v. 2.3.4), using very sensitive matching but requiring full end-to-end matches, with a maximum genomic match length of 1 kbp (bowtie2 options: –very-sensitive –phred33 –fr –end-to-end –maxins 1000 –minins 0). Mapped reads were filtered to remove those that could not be uniquely mapped to a single location, using samtools (v. 1.9) to exclude read pairs with a mapping quality (MAPQ) lower than 3 (samtools options: samtools view -f 2 -q 3).

Bigwig tracks for genomic signal correlation between samples and for visualization in genome browsers were generated using a combination of the UCSC Kent utilities (v. 385) genomeCoverageBed (options: -bg -pc -split -scale $SCALE) and bedGraphToBigWig. The $SCALE scaling factor was calculated as 1,000,000/total_mapped_reads, to normalize the coverage scores across samples with different sequencing depths. Duplicate reads were removed using Picard tools (v. 2.20) MarkDuplicates with the “REMOVE_DUPLICATES = TRUE” flag set.

### Global RNA and input DNA sample correlation

For calculating RNA and DNA global signal correlation, the BAC-covered genomic regions were divided into 1 kilobasepair bins, and the mean normalized bigwig signal in each bin was calculated using the "summary" function in the rtracklayer Bioconductor package^54^. Pearson correlation coefficients were calculated using the R "cor" function, and plotted using the "ggpairs" function in the GGally R package. RNA/DNA log2 fold change genomic signal bigwig tracks were generated using the DeepTools (v. 3.1) bigwigCompare command (options: –skipNonCoveredRegions –operation log2 –outFileFormat bigwig), and correlated and plotted in the same way as the RNA and DNA signal tracks above.

### Global coverage depth analysis

RNA and DNA global coverage levels across the BAC-covered regions was calculated using the bedtools (v2.27.1) “coverage” command, with the “-hist” option. Cumulative coverage calculation and plotting were performed in R, based on the histogram for the full set of BAC regions (the “all” histogram data in the bedtools coverage results).

### STARR-seq vs validation scores correlation

STARR-seq scores of the selected validation sequence genomic regions in Fig. 3 were calculated as described above for the global correlation between samples of the RNA/DNA log2 fold change genomic signal. The 1 kilobasepair genomic bins were replaced with the 9 validation sequence genomic regions. The mean bigwig signal for the RNA/DNA log2 fold change bigwig tracks in these regions was used, calculated using the "summary" function in the rtracklayer Bioconductor package. Validation scores were calculated as the median log10-transformed ratio of the normalized fluorescence signal in the validation sequence channel relative to the fluorescence signal of the CMV promoter control channel, across all cells in the assessed fields of view. The Pearson correlation coefficient between STARR-seq and validation scores was calculated using the R "cor" function.

### Peak calling

Candidate enhancers were identified using peak calling with the STARRPeaker program^34^ (v. 1.0, obtained from “https://github.com/gersteinlab/starrpeaker” on 22 March 2021 and modified to run with Python 3). A sliding window of 500 bp with step size 100 bp was used, and RNA secondary structure potential and GC-content as covariates. RNA secondary structure potential of the BAC-covered genomic regions was performed using LinearFold^55^ (obtained from “https://github.com/LinearFold/LinearFold” on 22 March 2021). GC content for the mouse mm10 genome assembly was downloaded from the UCSC Genome Browser website. The three merged technical replicates of the AAV-packaged input DNA library were used as input control. Merging was done by concatenating the read 1 and read 2 FASTQ files separately, maintaining the same order for each, and then running the mapping, filtering and deduplication steps as for the individual samples. For each RNA sample, peaks were called separately for each of the three AAV-packaged input DNA technical replicate input controls. The resulting peaks were merged across input control replicates, and only those detected in more than one replicate were retained. Peaks common to all five processed RNA samples were used as the final peak set.

For MACS2 peak calling, MACS2 (v. 2.1.2)^56^ was used without removal of duplicate reads (–keep-dup all), but with otherwise default settings. The three merged technical replicates of the AAV-packaged input DNA library were used as input control. Merging was done by concatenating the read 1 and read 2 FASTQ files separately, maintaining the same order for each, and then running the mapping, filtering and deduplication steps as for the individual samples.

### Shuffled control peak sets

1000 shuffled control peak sets were generated for the merged peak set using the bedtools (v. 2.27.1) “shuffle” command, restricting the peaks to the BAC-covered genomic regions (bedtools options: -noOverlapping -maxTries 10,000 -incl BAC_regions.bed -seed < square_of_trial_number >). For reproducibility, the random number generator for each trial was seeded with the square of the trial number.

### Evolutionary conservation of peaks

Evolutionary conservation of peaks was calculated based on the 60-way vertebrate conservation Phastcons bigwig track from the UCSC Genome Browser database (mm10.60way.phastCons.bw). The bigWigAverageOverBed tool from the UCSC Genome Browser ‘s Kent utilities (v. 365) was used to calculate the average score per peak.

### Peak overlap with epigenetic signatures

Open chromatin datasets (BED-format files) were downloaded from the mouse ENCODE project (cortex DnaseI Hypersensitive Sites from 8 week mice, 3 replicates, IDs: ENCFF413ZWP, ENCFF673QYR & ENCFF359OPB). ENCODE DHS replicates were merged using bedtools merge. Enhancer-associated histone mark datasets for mouse brain were also downloaded from the mouse ENCODE project (H3K27ac & H3K4me1, IDs: ENCFF199XZU & ENCFF354KFL respectively)^15,35^. Overlap of the merged STARR-seq peaks with the various mouse brain epigenetic datasets was performed using the “countOverlaps” function of the GenomicRanges Bioconductor package. Epigenetic signal heatmaps were created using the EnrichedHeatmap Bioconductor package^57^.

### Peak overlap with repetitive element classes

Repetitive element genomic locations for mouse genome assembly mm10 were taken from the repeatmasker table dump file (rmsk.txt.gz) downloaded from the UCSC Genome Browser. The different repeat classes were filtered based on the “repClass” field. Overlap with the merged STARR-seq peaks was performed using the “countOverlaps” function of the GenomicRanges Bioconductor package.

### Plotting, image and statistical analyses

Where not otherwise specified, plotting and statistical analyses were performed using the R statistical analysis program^58^ (v. 4.0.3), including packages from the Bioconductor project^59^ (v. 3.12). Plots in Figs. 1, 2 and 3 and supplemental Figs. S1, S2 and S3 were done using the ggplot2 R package^60^ (v. 3.3.3), except for the epigenetic signal heatmaps. Plots in Figs. 4, 5, 6 and 7 were done using Matlab (v. R2016b). Image analyses in Figs. 4, 5, 6 and 7 were performed with Fiji (v. 1.53c), unless otherwise specified.

### Preparation of histological samples

The brains were extracted after 3 weeks of post-injection incubation. Mice were deeply anesthetized with ketamine/medetomidine mixture and isoflurane and transcardially perfused with PBS followed by 4% paraformaldehyde (PFA) in PBS. The brains were post-fixed in 4% PFA overnight at 4 °C. Slices of 70 µm or 500 µm (for cell tracing) coronal section were obtained using a Leica VT1000S vibratome.

Nuclei were stained with DAPI and slices were mounted in Fluoromount-G. Slices of 500 µm thickness were cleared with CUBIC reagent as follows: To delipidate, sections were washed with PBS for 2 h at room temperature and treated with 50% CUBIC-L overnight at 37 °C and further with 100% CUBIC-L for 1 day at 37 °C. Sections were subsequently washed with PBS for 2 h at room temperature and nuclei were stained with DAPI. To match RI, sections were treated with 50% CUBIC-R + overnight at room temperature and then with 100% CUBIC-R + for 1–2 days at room temperature. Sections were mounted in 100% CUBIC-R + using Gene Frames (Thermo Scientific).

### Immunostaining

Slices were permeabilized in blocking buffer (10% normal goat serum and 1% TritonX-100 in PBS) for 2 h at room temperature and washed three times with PBS. Antibodies were prepared in 1% blocking buffer: biotin-conjugated anti-NeuN at 1:1000 (Merck, MAB377B); mouse anti-parvalbumin at 1:2000 (Swant, PV 235); rabbit anti-calbindin at 1:2000 (Swant, CB38); rabbit anti-Olig2 at 1:1000 (Merck, AB9610); streptavidin Alexa Fluor 647, goat anti-mouse Alexa Fluor 647, and goat anti-rabbit Alexa Fluor 555 at 1:500, (Thermo Fisher, S32357, A21235, A21428). Slices were incubated in primary antibodies overnight at 4 °C and washed three times in PBS. Slices were then incubated in secondary antibodies for 2 h at room temperature and washed three times in PBS. Nuclei were stained with DAPI.

### Confocal imaging

Images of 70 um slices were taken with a 40 × oil objective using a Leica TCS SP5 confocal microscope. Crosstalk of multiplexed fluorescence images were corrected using Leica TCS SP5 linear unmixing. For imaging of multiplexed fluorescence VS construct, lasers 405, 458, 488, 514, and 561 were used for DAPI, mTFP, GFP, Venus, and tagRFP and the images were taken sequentially. For imaging of three CTB fluorescence conjugates, lasers 514, 561, and 647 were used for alexa fluor 488, alexa fluor 555, and alexa fluor 647. Laser 514 is used to minimize the crosstalk from mTFP. Images of 500 um slices were taken with a 20 × water objective using a Visitron spinning disc confocal microscope. Laser 405 and 515 were used for DAPI and EYFP.

### Image analysis

For segmentation of individual cells, the DAPI signal was pre-processed with ImageJ median filter for signal smoothing and convolution for boundary sharpening. Segmentation of individual nuclei was then performed using the ImageJ plugin StarDist and resulting ROIs were used to measure fluorescence signals from each channel as mean intensity. Mean intensity outside of ROIs was used to estimate the background signal. Signals in each channel were classified as positive if the signal exceeded the background signal plus three times its Standard deviation.

For cell reconstruction, cells from 500 um slice were semi-automatically traced using ImageJ plugin SNT.

## Supplementary Information

Supplementary Information.
