# Supplementary material for: An unbiased AAV-STARR-seq screen revealing the enhancer activity map of genomic regions in the mouse brain in vivo
Source: Sci Rep. 2023 Apr 25;13:6745. doi: 10.1038/s41598-023-33448-w (PMC10130037; doi:10.1038/s41598-023-33448-w)
Supplement: Supplementary file 1 — Supplementary Information. [file 41598_2023_33448_MOESM1_ESM.pdf]

Supplementary Figures and Tables

An unbiased AAV-STARR-seq screen revealing the enhancer activity map of genomic regions in the mouse brain *in vivo*.

Ya-Chien Chan[1,9], Eike Kienle[1,9], Martin Oti[2,6,9], Antonella Di Liddo[2], Maria Mendez-Lago[2], Dominik F. Aschauer[1], Manuel Peter[3,7], Michaela Pagani[3], Cosmas Arnold[3,8], Andreas Vonderheit[2], Christian Schön[4], Sebastian Kreuz[4], Alexander Stark[3,5], Simon Rumpel[1,10*]

1: Institute of Physiology, Focus Program Translational Neurosciences, University Medical Center, Johannes Gutenberg University Mainz, Mainz, Germany
2: Institute of Molecular Biology GmbH (IMB), Mainz, Germany
3: Research Institute of Molecular Pathology (IMP), Vienna Biocenter (VBC), Vienna, Austria
4: Research Beyond Borders, Boehringer Ingelheim Pharma GmbH & Co. KG, Biberach an der Riß, Germany
5: Medical University of Vienna, Vienna BioCenter (VBC), 1030 Vienna, Austria
6: current address: Global Computational Biology and Digital Sciences, Boehringer Ingelheim Pharma GmbH & Co. KG, Biberach an der Riß, Germany
7: current address: Department of Stem Cell and Regenerative Biology, Harvard University, Cambridge, Massachusetts, United States of America
8: current address: CeMM Research Center for Molecular Medicine of the Austrian Academy of Sciences, Vienna, Austria
9: These authors contributed equally
10: Lead contact: sirumpel@uni-mainz.de
*Correspondence: sirumpel@uni-mainz.de

Supplementary Figure 1:

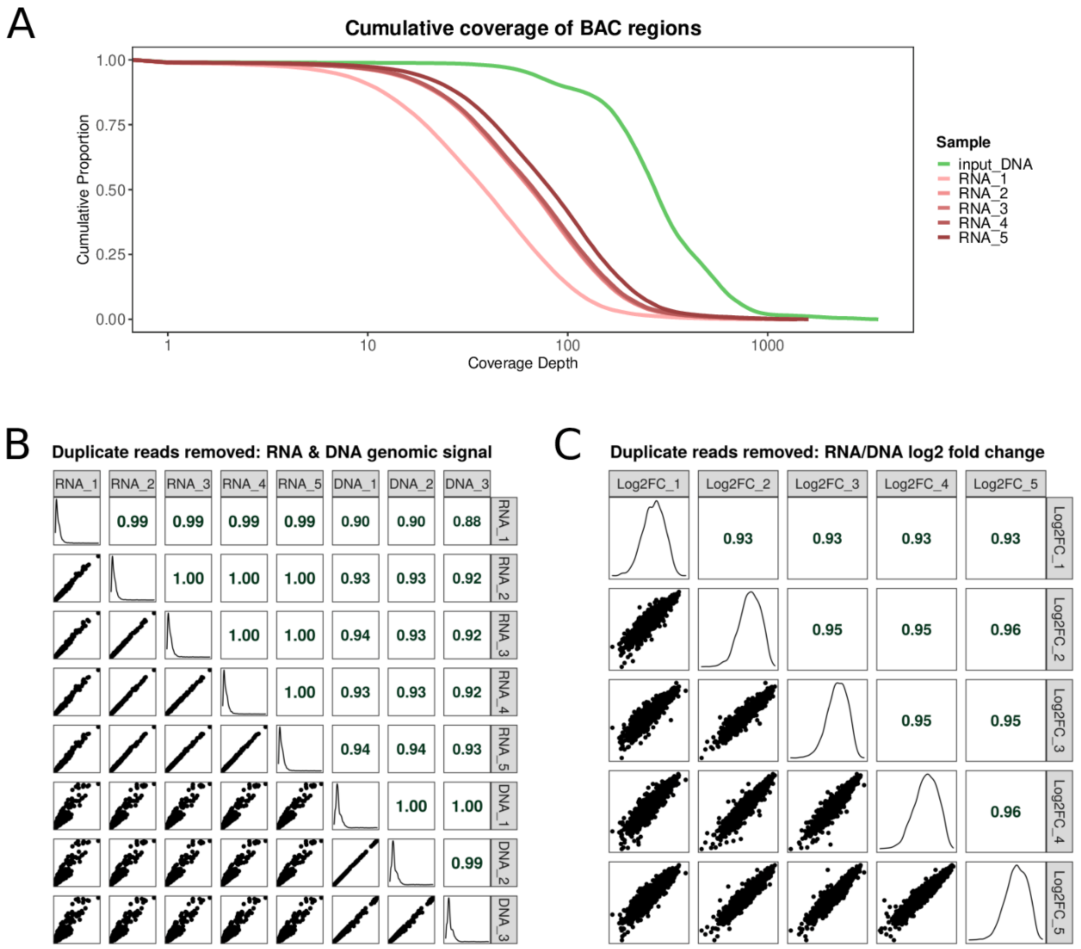

AAV-STARR-seq screen sequencing results. (**A**) Coverage of the mouse BAC-covered genomic regions. (**B**) Deduplicated reads correlogram with Pearson correlation coefficients for all mouse RNA biological replicates and input library DNA technical replicates, based on 1 kb bins tiled across the BAC regions. The diagonal plots show the expression distributions of the 1 kb bins per sample. RNA samples stem from the independent screens in individual mice. DNA samples are technical replicates of the AAV-STARR-seq screening library used as input. (**C**) Deduplicated reads correlogram with Pearson correlation coefficients for the STARR-seq signal (RNA/input DNA log2 fold change) across all mice.

Supplementary Figure 2:

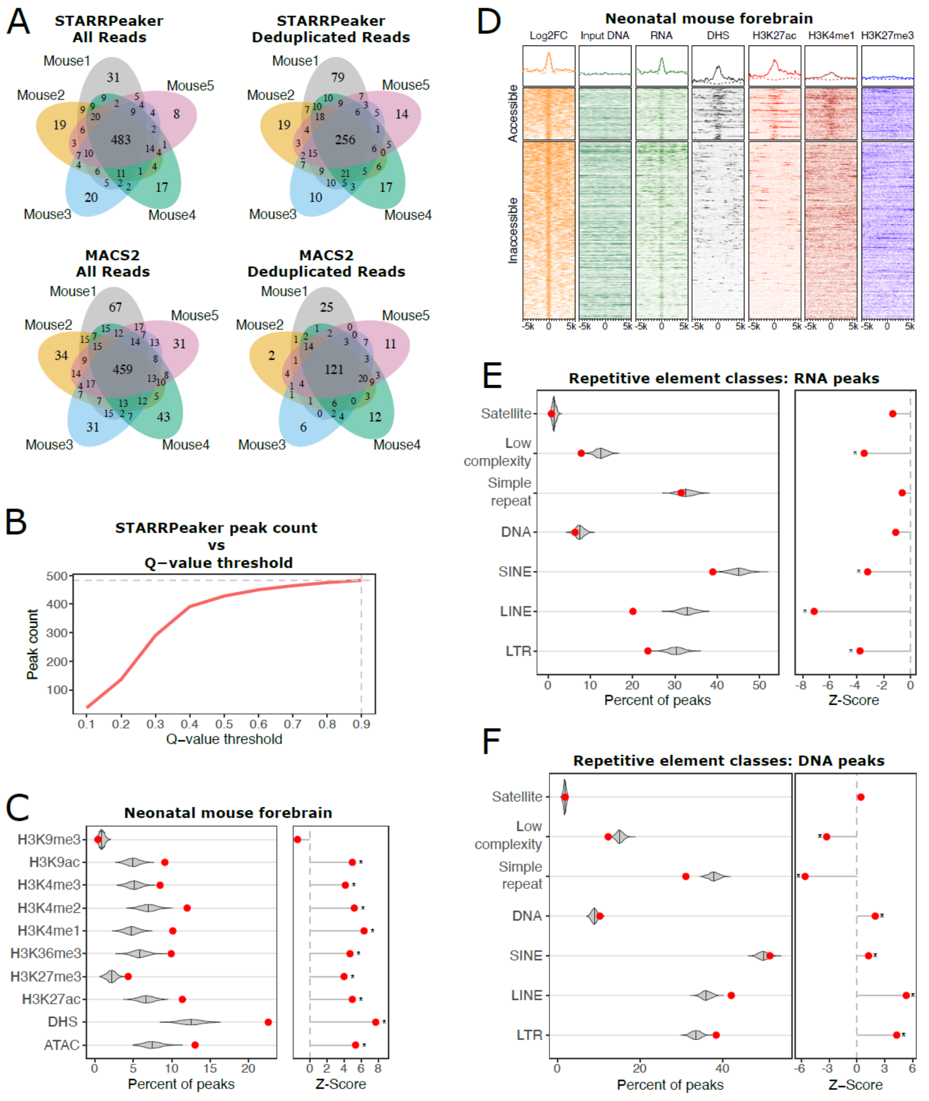

Extended analysis of candidate enhancer identification. (**A**) STARR-seq peak reproducibility across replicates for different peak calling strategies. Shown are Venn diagrams for the peaks identified in the five individual mice. The four plots show data for (left to right): STARRPeaker peaks using all sequenced reads (top left), STARRPeaker peaks using deduplicated reads (top right), MACS2 peaks using all sequenced reads (bottom left), MACS2 peaks using deduplicated reads (bottom right). (**B**) STARRPeaker peak counts at different Q-value thresholds. (**C**) Overlap of STARR-seq peaks with various histone marks and accessible chromatin regions in postnatal mouse brain cortex (red dots), compared with 1000 shuffled controls (violin plots). The right panel lollipop plot shows the Z-scores of the real overlap based on fitting a normal distribution to the random controls. Asterisks indicate significant differences based on empirical p<0.05. All datasets are from the ENCODE project. DHS: Dnase1 Hypersensitive Sites; ATAC: ATAC-seq peaks. (**D**) Heatmap of STARR-seq and postnatal mouse brain histone mark signal around STARR-seq peaks. The heatmap is clustered into STARR-seq peaks overlapping accessible chromatin peaks (above) and non-overlapping peaks (below), and sorted by input DNA level. (**E**) Overlap of STARR-seq peaks with different classes of repetitive elements. Asterisks indicate significant differences based on empirical p<0.05. (**F**) Overlap of DNA-over-RNA peaks (RNA-depleted regions) with different classes of repetitive elements. Asterisks indicate significant differences based on empirical p<0.05.

Supplementary Figure 3:

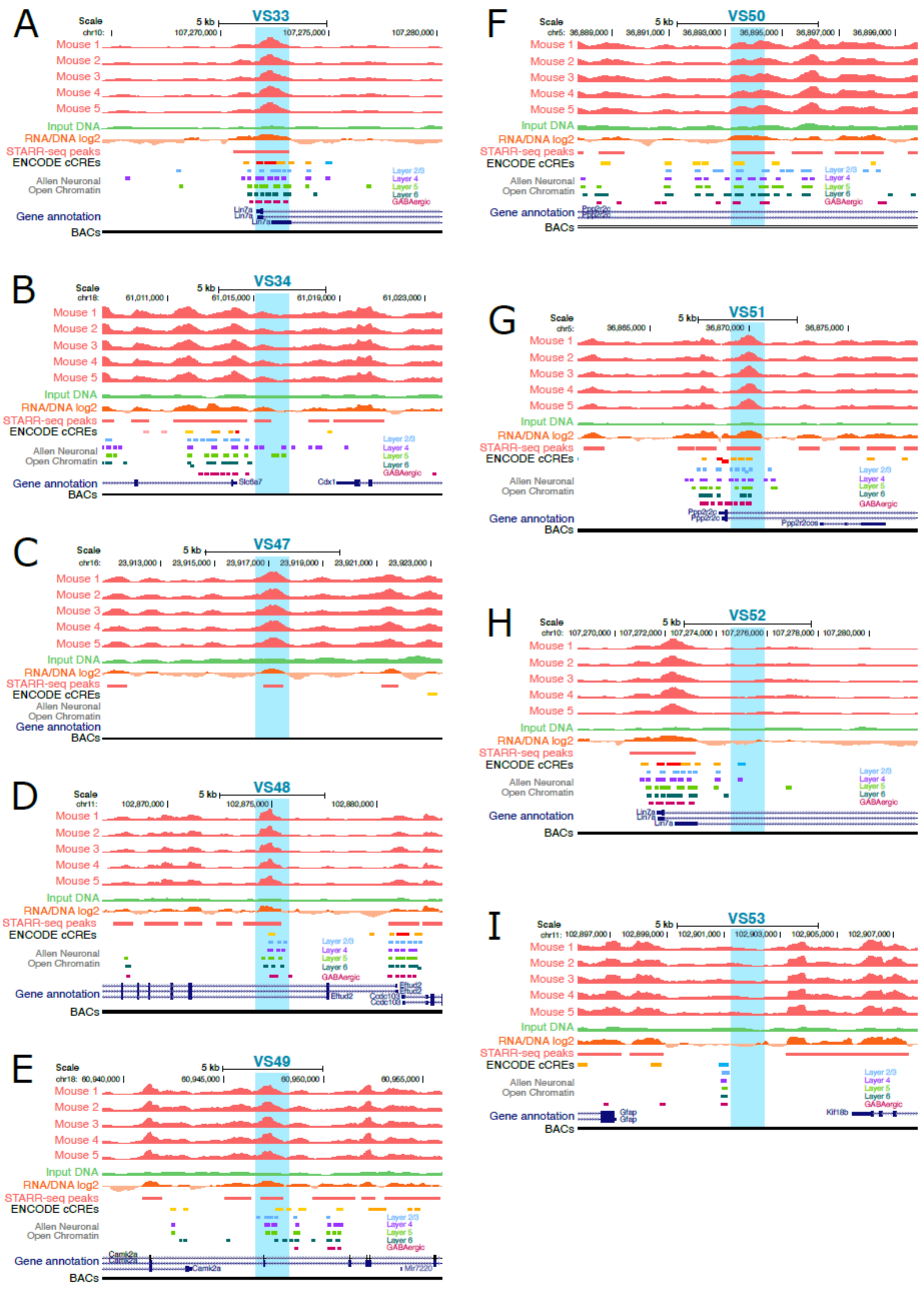

Description of the screened genomic regions selected for validation: UCSC genome browser screenshots of the 9 regions (A-I) of varying predicted enhancer activity used for the multicolor fluorescence microscopy validation experiment. The tracks are (top to bottom): STARR-seq RNA for the 5 mice, merged input DNA, merged RNA / merged input DNA log2 fold change signal, STARRPeaker-called peaks, ENCODE project cis-regulatory elements (red: promoter, orange: enhancer, blue: insulator), cortical layer-specific and GABAergic neuron ATAC-seq peaks from the Allen Brain Institute, and gene annotation. Blue highlighted regions indicate the sequences chosen for further analysis.

Supplementary Table 1:

BACs utilized in this study

BAC clone	Chromosome	Start	End	BAC size (kb)	Target gene
**RP23-100J13**	chr10	107113919	107310064	196.145	Lin7a
**RP23-463E7**	chr11	102829076	103027447	198.371	Gfap
**RP23-306D19**	chr12	38595495	38791798	196.303	Etv1
**RP24-357G23**	chr12	38681492	38836545	155.053	Etv1
**RP23-112H14**	chr12	38816973	39036494	219.521	Etv1
**RP24-403D23**	chr16	23798011	23944445	146.434	Sst
**RP23-274H19**	chr16	23879506	24077296	197.790	Sst
**RP23-385E10**	chr18	60715061	60915371	200.310	Camk2a
**RP24-243J21**	chr18	60883184	61026229	143.045	Camk2a
**RP24-211A8**	chr18	60977513	61136779	159.266	Camk2a
**RP23-407K8**	chr2	22551742	22758153	206.411	Gad2
**RP23-114M13**	chr2	22702598	22920596	217.998	Gad2
**RP23-373H20**	chr5	36741605	36905617	164.012	Wfs1
**RP23-16N23**	chr5	36880272	37074653	194.381	Wfs1
**RP23-106D12**	chr5	37022507	37215239	192.732	Wfs1
**RP23-346H23**	chr6	85116745	85346354	229.609	Emx1
**RP23-275N2**	chrX	20911235	21111402	200.167	Syn1

Supplementary Table 2:

Sequencing results of RNA samples from the independent screens in individual mice and the three DNA samples taken as technical replicates from the input screening library

Sample	Total sequenced read pairs	Uniquely mapped read pairs	Multi-mapped read pairs	Discordantly mapped read pairs	Uniquely mapped single reads	Multi-mapped single reads	Overall alignment rate	Distinct uniquely mapped read pairs
**RNA_1**	18,412,017 (100.00%)	12,523,870 (68.02%)	639,570 (3.47%)	68,529 (0.37%)	2,559,689 (13.90%)	231,242 (1.26%)	79.45%	296,327
**RNA_2**	16,139,538 (100.00%)	10,893,731 (67.50%)	556,380 (3.45%)	66,212 (0.41%)	2,227,691 (13.80%)	197,231 (1.22%)	78.87%	472,017
**RNA_3**	14,955,083 (100.00%)	10,093,066 (67.49%)	501,608 (3.35%)	57,575 (0.38%)	2,001,040 (13.38%)	176,119 (1.18%)	78.51%	479,501
**RNA_4**	15,188,697 (100.00%)	10,241,300 (67.43%)	535,980 (3.53%)	69,478 (0.46%)	2,158,585 (14.21%)	197,275 (1.30%)	79.17%	490,772
**RNA_5**	19,346,587 (100.00%)	12,973,369 (67.06%)	676,780 (3.50%)	87,396 (0.45%)	2,624,360 (13.56%)	233,021 (1.20%)	78.39%	583,474
**RNA_6**	1,159,987 (100.0%)	786,764 (67.8%)	40,349 (3.5%)	4,668 (0.4%)	159,531 (13.8%)	14,296 (1.2%)	79.20%	292,722
**DNA_1**	12,893,259 (100.00%)	10,449,978 (81.050%)	634,706 (4.923%)	10,879 (0.084%)	218,942 (1.698%)	37,512 (0.291%)	87.05%	1,368,800
**DNA_2**	15,230,837 (100.00%)	12,393,265 (81.370%)	762,678 (5.007%)	13,335 (0.088%)	253,875 (1.667%)	44,569 (0.293%)	87.44%	1,457,255
**DNA_3**	9,347,247 (100.00%)	7,549,739 (80.770%)	485,494 (5.194%)	6,020 (0.064%)	143,515 (1.535%)	25,023 (0.268%)	86.93%	1,183,900

* Sample RNA_6 was excluded from further analysis as the number of sequenced and mapped reads was less than 10% of the other RNA samples.
